# SARS-CoV-2 nonstructural protein 1 suppresses host transcription by reducing RNA polymerase II levels

**DOI:** 10.1016/j.isci.2025.114233

**Published:** 2025-11-26

**Authors:** Jianfang Li, Kang Wang, Jie Wang, Caizhou Zhong, Shenglan Wang, Hong Peng, Zheng Li

**Affiliations:** 1 Department of Basic Research, Guangzhou National Laboratory, Guangzhou 510005, China; 2 School of Basic Medical Sciences, Guangzhou National Laboratory, Guangzhou Medical University, Guangzhou 510005, China; 3 State Key Laboratory of Medicinal Chemical Biology, Tianjin Key Laboratory of Protein Science, and College of Life Sciences, Nankai University, 94 Weijin Road, Tianjin 300071, China; 4 Shenzhen Key Laboratory of Systems Medicine for Inflammatory Diseases, Centre for Infection and Immunity Study (CIIS), School of Medicine, Shenzhen Campus of Sun Yat-sen University, Shenzhen 518197, China

**Keywords:** Molecular biology

## Abstract

The COVID-19 pandemic has caused devastating global losses and massive mortality. The nonstructural protein 1 (NSP1) of SARS-CoV-2 plays a vital role in suppressing host protein synthesis. However, its effect on host gene transcription remains uncertain. We established a reporter system that enables the isolation of NSP1-overexpressing cells and confirmed that NSP1 leads to a global reduction in host mRNA levels, including those of common housekeeping genes. To accurately quantify transcriptomic changes in total and nascent RNA, we developed a pipeline integrating thio-labeled RNA sequencing (SLAM-seq) with External RNA Controls Consortium (ERCC) spike-in RNA normalization. Our research revealed a widespread downregulation of host mRNAs upon NSP1 expression, with virtually no genes significantly upregulated. Normalizing nascent RNA with ERCC spike-ins illustrated that NSP1 obstructed transcription by reducing RNA polymerase II levels. This study establishes a robust framework for investigating NSP1 and viral factors, providing insights into coronavirus-host interactions and potential therapies.

## Highlights

• SLAM-seq with spike-in normalization enables precise RNA quantification
• NSP1 reduces host nascent RNAs globally
• NSP1 lowers RNA polymerase II to block host transcription

## Graphical abstract

**Figure:**
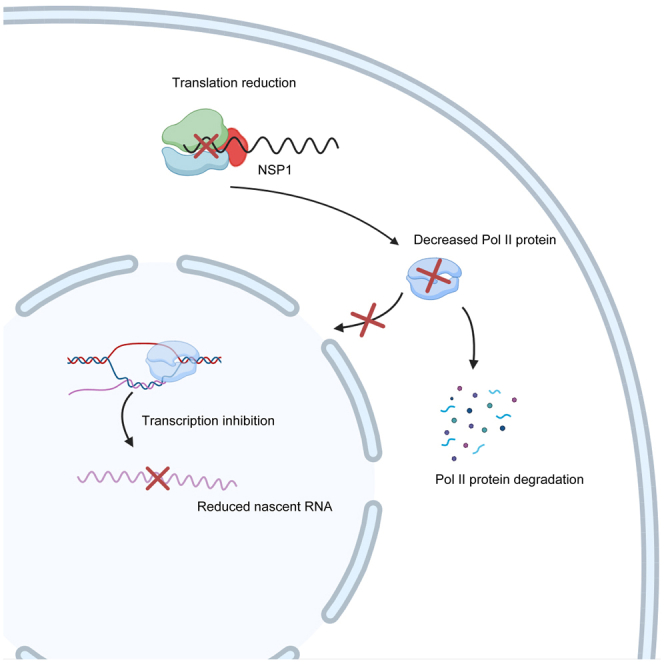

## Introduction

The outbreak of the novel coronavirus SARS-CoV-2, which causes coronavirus disease 2019 (COVID-19) worldwide pandemic and millions of deaths, has had profound and far-reaching effects on global public health, society, and the economy.^1^^,^^2^ While the global health crisis triggered by COVID-19 has gradually abated due to mass vaccination efforts, the development of antiviral therapies, and improved public health strategies.^3^^,^^4^^,^^5^ Understanding how SARS-CoV-2 interacts with host cells is crucial for grasping COVID-19 pathogenesis and preparing for future outbreaks of similar coronaviruses.

Besides the main structural proteins, SARS-CoV-2 also encodes 16 nonstructural proteins (NSPs) that are essential for viral replication and host immune subversion.^6^^,^^7^^,^^8^ NSP1 is recognized as the primary viral shutoff factor and plays a pivotal role in suppressing host gene expression. Beyond its ability to inhibit host translation,^9^^,^^10^^,^^11^^,^^12^^,^^13^^,^^14^^,^^15^^,^^16^ one of the most striking functions of NSP1 is its capacity to induce extensive degradation of host mRNAs. Previous studies have shown that NSP1 recruits cellular nucleases or interferes with RNA stability pathways to trigger widespread mRNA decay, leading to a rapid loss of host transcripts.^11^^,^^12^^,^^13^^,^^14^^,^^17^^,^^18^^,^^19^^,^^20^ This activity not only diminishes the availability of cellular mRNAs for translation but also broadly reprograms the transcriptome to suppress antiviral responses. In parallel, NSP1 blocks nuclear mRNA export,^11^^,^^21^ further restricting transcript availability. These findings elucidate the intricate interactions of NSP1 with the post-transcriptional regulation of mRNAs. However, its potential role in modulating mRNA synthesis remains unclear. Uncovering NSP1’s transcriptional regulatory mechanism represents a possible approach for developing novel NSP1-specific inhibitors, thus paving the way for new therapies against SARS-CoV-2 and other circulating and emerging coronavirus infections.

Gene expression is a dynamic biological process governed by the intricate interplay of RNA transcription, elongation, and decay.^22^ While RNA sequencing (RNA-Seq) has emerged as a standard tool for quantifying total RNA abundance and has been extensively employed in prior studies to investigate the effects of NSP proteins in host cells, its utility in elucidating transcriptional regulation remains constrained.^23^ The cellular abundance of mRNA is determined by both transcriptional synthesis and mRNA degradation.^24^ Although total mRNA levels often reflect transcriptional activity, this correlation can be unreliable in cells where mRNA degradation is particularly active. In such cases, total mRNA levels may not accurately represent the true transcription rate. Therefore, alternative approaches are required to directly assess transcriptional activity within cells. To precisely discover the NSP1’s regulatory function on host gene transcription, we adopt the thio-labeled technique SLAM-seq (thiol (SH)-linked alkylation for metabolic sequencing) to quantitatively assess transcription levels by specifically metabolically labeling newly transcribed mRNAs.^25^ Through the conversion of 4-thiouridine (4sU) to cytosine utilizing iodoacetamide, the samples undergo sequencing without prior separation. This methodology facilitates the identification of newly synthesized RNA by exploiting specific T to C mismatches in the reads aligned to the reference transcriptome, thereby differentiating it from pre-existing, unlabeled RNA. Additionally, by concurrently quantifying both nascent and total RNA fractions, this approach has the potential to analyze transcriptional dynamics and RNA stability in a parallel manner.

Furthermore, traditional normalization methods employed in RNA-Seq analyses predominantly consider sequencing depth to ensure the total number of sequenced reads remains consistent across all samples. This methodology assumes that the total mRNA quantity from each experimental condition is invariant. Nevertheless, this assumption does not hold true for cells transfected with NSP1, which is well-known for its capability to degrade host cytosolic cellular mRNAs. Consequently, significant discrepancies in the levels of total mRNA have been observed between transfected and untransfected cells.^11^ To resolve this quantification issue, External RNA Controls Consortium (ERCC) spike-in controls,^26^ which consist of 92 synthetic RNAs with defined concentrations, can be used for the precise interpretation of potential alterations in gene expression levels across samples.

In this study, we constructed a cell reporter system to isolate NSP1 overexpressed cells to elucidate the role of NSP1 in mRNA regulation, then we implemented 4sU labeling and incorporated ERCC spike-in controls to delineate the transcriptional regulatory function of NSP1. The analysis results indicated that, in addition to its effects on post-transcriptional processes, NSP1 significantly inhibited the transcriptional activity of numerous protein-coding genes. Furthermore, the inhibition of transcription was related to the interference of the NSP1 protein with RNA polymerase II. The detrimental effects of the NSP1 protein following SARS-CoV-2 infection appear to be more pronounced than previously understood. The dual inhibition effect of the NSP1 protein on both the transcriptional and translational processes may represent potential drug targets. Overall, such findings could uncover novel strategies employed by SARS-CoV-2 to modulate gene expression at multiple levels, including transcription and translation, which help open new avenues for therapeutic intervention.

## Results

### Establishment of NSP1 overexpressed cells

**Figure 1:**
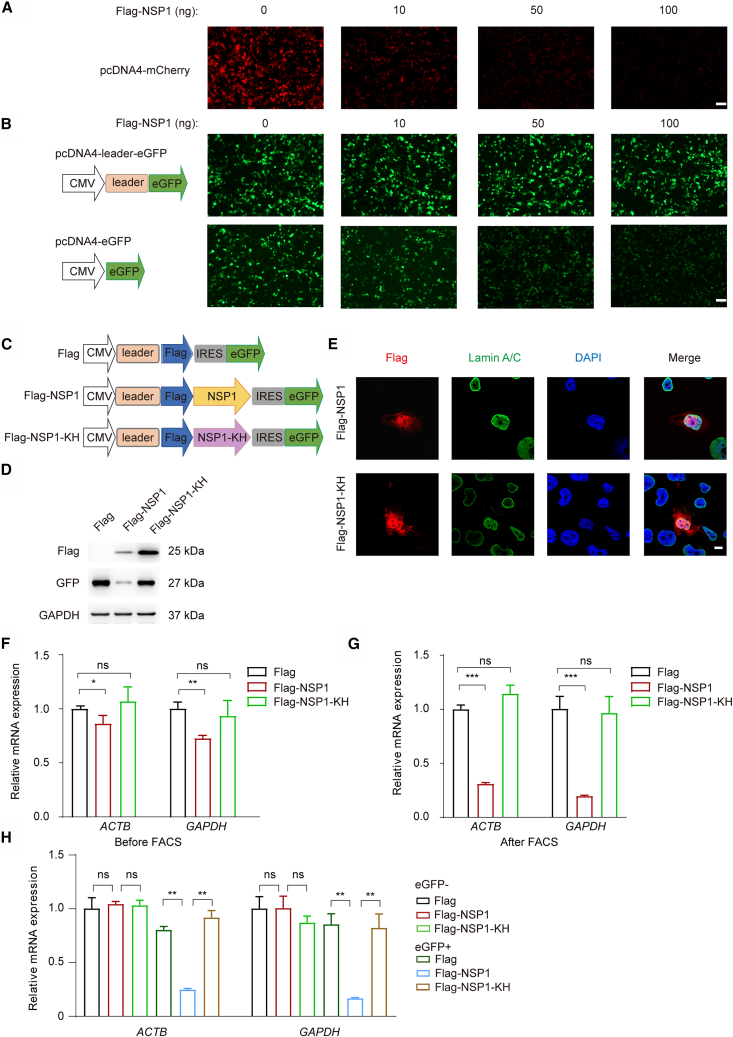
NSP1 protein impeded the gene expression of housekeeping genes (A) Analysis of mCherry expression in 293T cells co-transfected with mCherry plasmids and increasing amounts of Flag-NSP1 plasmids. Scale bars, 100 μm. (B) Analysis of GFP expression in 293T cells transfected with eGFP plasmids, with or without the SARS-CoV-2 leader sequence, in the presence of increasing amounts of Flag-NSP1 plasmids. Scale bars, 100 μm. (C) Schematic representation of Flag-NSP1 overexpression constructs. The SARS-CoV-2 leader sequence was inserted downstream of the promoter and upstream of the Flag-NSP1 coding sequence, followed by IRES-eGFP sequence. (D) Western blot analysis of GAPDH, eGFP, and Flag-NSP1 protein levels in H1299 cells transfected with Flag, Flag-NSP1, or Flag-NSP1-KH plasmids. Antibodies used are indicated on the right. (E) Immunofluorescence analysis of H1299 cells transfected with Flag-NSP1 or Flag-NSP1-KH plasmids. NSP1 proteins were detected using anti-Flag antibody (red), the nuclear envelope marker Lamin A/C (green), and DAPI (blue). Scale bars, 10 μm. (F and G) RT-qPCR analysis of *ACTB* and *GAPDH* mRNA levels in H1299 cells transfected with the indicated plasmids before (F) or after (G) FACS sorting for eGFP-positive cells. Expression levels were normalized to 18S rRNA and presented relative to Flag control (set as 1; *n* = 3 per group; mean ± SD; Student’s *t* test; ∗*p <* 0.05; ∗∗*p <* 0.01; ∗∗∗*p <* 0.001; ns, no significance). (H) RT-qPCR analysis of *ACTB* and *GAPDH* mRNA levels in eGFP-positive and eGFP-negative H1299 cells transfected with the indicated plasmids. Expression levels were normalized to 18S rRNA and presented relative to Flag control in eGFP-negative cells (set as 1; *n* = 3 per group; mean ± SD; Student’s *t* test; ∗∗*p <* 0.01; ns, no significance).

To investigate the functional role of NSP1 protein in host cells, we constructed the expression plasmids encoding NSP1 and NSP1-KH (K164A/H165A) mutant with an N-terminal Flag tag for western blotting and co-immunoprecipitation (Co-IP) detection, as well as those plasmids with IRES-eGFP elements for cell sorting. We co-expressed Flag-NSP1 and mCherry in 293T cells and observed a dose-dependent reduction in mCherry fluorescence (Figure 1A). This suggests that NSP1 not only suppresses host mRNA translation and promotes degradation of cytosolic transcripts as previously reported^11^ but also downregulates CMV-driven ectopic gene expression. Notably, as N-terminal tags may potentially interfere with protein function, we also produced an untagged version of NSP1. Importantly, the Flag-tagged NSP1 exhibited a similar inhibition function to the untagged protein (Figure S1A), confirming that the tag does not impair its function. These findings imply that NSP1 may self-limit its expression in host cells.

To get stable expression of NSP1, we constructed plasmids containing the SARS-CoV-2 5′ leader sequence.^20^ When positioned directly adjacent to the CMV promoter, the leader sequence significantly enhanced eGFP expression and translation (Figure 1B). This result indicates that proper positioning of the leader sequence is critical for mRNA stability and translational efficiency under NSP1-mediated suppression.

Based on these findings, we engineered a chimeric construct in which the SARS-CoV-2 5′ leader was fused upstream of the NSP1 coding sequence to mitigate self-repression and allow robust NSP1 expression (Figure 1C). As well as the double mutant, NSP1-KH (K164A/H165A), which is deficient in 40S ribosomal subunit binding and has lost its ability to repress host mRNA translation,^13^ to serve as a negative control. An empty Flag vector was used as an additional control. Following transfection into H1299, western blot analysis confirmed the expression of both NSP1 and NSP1-KH protein (Figure 1D). In addition, we observed a reduction in GFP protein levels in cells overexpressing NSP1, likely due to NSP1 highly expressed cells dying under strong transcriptional repression (Figure 1D), which has been reported in prior research.^9^ We further investigated the subcellular localization of NSP1 by immunofluorescence. Notably, both NSP1 and NSP1-KH were detected in the cytoplasm and the nucleus (Figures 1E and S1B). The cytoplasmic distribution is consistent with NSP1’s known function in binding ribosomes and inhibiting host mRNA translation.^9^^,^^10^^,^^11^^,^^12^^,^^13^^,^^14^^,^^15^^,^^16^ Interestingly, the nuclear localization of NSP1 suggests an additional role in regulating host transcription. These observations support that NSP1 may act at multiple levels, both transcriptional and translational, to reprogram host gene expression during SARS-CoV-2 infection.

### NSP1 impeded the expression of housekeeping genes

As shown in Figure 1E, only a portion of H1299 cells expressed NSP1 following lipid-based transfection. However, to exclude confounding effects from untransfected cells, we used fluorescence-activated cell sorting (FACS) to isolate eGFP-positive cells. FACS analysis showed comparable proportions of eGFP-positive cells across all transfection groups (Figure S1C), indicating consistent transfection efficiency not impaired by NSP1.

Importantly, only minor changes in *GAPDH* and *ACTB* expression were detected in Flag-NSP1-transfected cells prior to sorting (Figure 1F); however, FACS-sorted NSP1-expressing cells exhibited a significant reduction in both genes (Figure 1G). Furthermore, both *GAPDH* and *ACTB* expression remained unchanged in eGFP-negative cells (Figure 1H), confirming that the observed transcriptional repression was specific to NSP1-expressing cells. To further elucidate the inhibitory function of NSP1, we conducted experiments using untagged NSP1-transfected cells, and the results corroborated its inhibitory effect on housekeeping genes (Figure S1D). These findings indicate that NSP1 not only impairs translation but also significantly influences host mRNA transcription. Given the strong repressive activity of NSP1, analysis of unsorted cell populations could obscure its true transcriptional impact. Thus, isolating positively transfected cells via FACS is critical for obtaining accurate and biologically meaningful transcriptomic data.

### A novel strategy to investigate NSP1-mediated transcriptional repression in host cells

**Figure 2:**
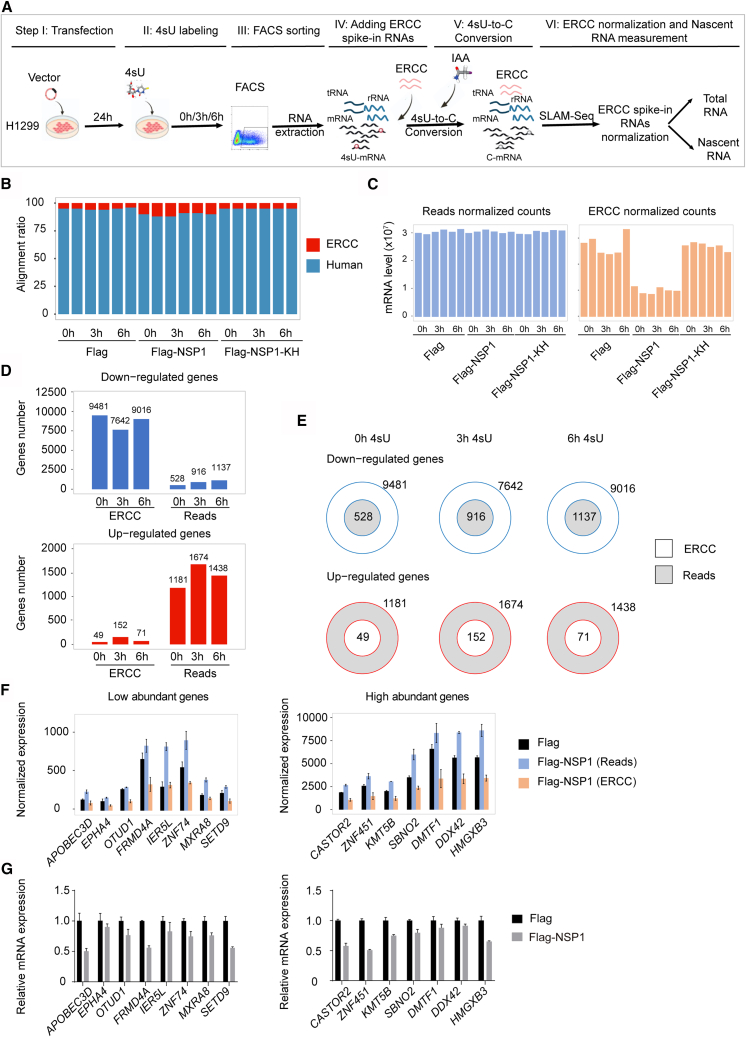
Strategy to investigate NSP1 effects on host RNA synthesis (A) Schematic illustration of the experimental workflow. H1299 cells were transfected with the indicated plasmids, labeled with 4sU for 0, 3, or 6 h, and eGFP-positive cells were sorted by FACS. IAA was used to convert 4sU to C, and ERCC spike-ins were added before library preparation (*n* = 2 per group). (B) Percentage of sequencing reads derived from H1299 cells (Human) or ERCC spike-ins across all samples. (C) Total mRNA levels normalized by sequencing reads or ERCC spike-ins. (D) Number of downregulated (top) and upregulated (bottom) DEGs identified using reads or ERCC normalization (Flag-NSP1 vs. Flag). (E) Venn diagrams showing overlap of DEGs identified using reads or ERCC normalization across all 4sU labeling times (Flag-NSP1 vs. Flag). (F) Expression levels of low-abundance (<1,000, left) or high-abundance (>1,000, right) genes normalized by reads or ERCC in Flag control (*n* = 2 per group; mean ± SD). (G) RT-qPCR validation of selected genes identified by SLAM-seq. Values were normalized to 18S rRNA and presented relative to Flag control (set as 1; *n* = 3 per group; mean ± SD).

Considering that the NSP1 decreased the mRNA levels of housekeeping genes such as *GAPDH* and *ACTB* (Figure 1G). We developed a novel strategy to evaluate transcriptomic changes in Flag-NSP1-transfected cells more accurately. This approach combines 4sU metabolic labeling with FACS-based isolation of transfected cells, as detailed in the “STAR Methods” section. By further integrating ERCC spike-in normalization, this strategy enables precise quantification of both total and nascent RNA, allowing for high-resolution analysis of transcriptional dynamics in host cells (Figure 2A).

Following FACS sorting, 22.9%–27.6% of the NSP1-overexpressed cells exhibited GFP-positive signals and were subsequently isolated for downstream analysis (Figure S1C). An equal amount of ERCC spike-in controls was added to each RNA sample. Notably, the number of sequencing reads derived from ERCC spike-in controls was significantly higher in NSP1-overexpressed cells than in those transfected with either Flag or Flag-NSP1-KH constructs (Figure 2B). This result confirms the mRNA-degrading activity of NSP1 and underscores the substantial reduction in endogenous mRNA abundance in NSP1-expressing cells. Under these conditions, external spike-in controls are critical for accurate normalization. Furthermore, normalized Fragments Per Kilobase of transcript per Million mapped reads (FPKM) values of each ERCC spike-in correlated strongly with their known input concentrations (R^2^ > 0.95) (Figure S1E), validating the consistency and quantitative reliability of the spike-in strategy for subsequent transcriptomic analyses.

Subsequently, we applied two normalization strategies, one using ERCC spike-in controls and the other based on total sequencing reads, to compare mRNA abundance and identify differentially expressed genes (DEGs) across samples. Notably, the two normalization methods yielded markedly different results. When normalized with ERCC spike-in controls, Flag-NSP1-transfected cells showed a pronounced reduction in mRNA abundance compared to either Flag- or Flag-NSP1-KH-transfected controls (Figure 2C), consistent with previous observations from SARS-CoV-2-infected Calu-3 cells.^11^^,^^20^ For a given vector, mRNA levels remain relatively stable across 4sU labeling time points. Specifically, in Flag-NSP1-transfected cells compared to Flag-transfected controls at 24, 27, and 30 h after transfection (corresponding to no labeling, 3- and 6-h 4sU treatments, respectively), the numbers of significantly downregulated genes were 9,481, 7,642, and 9,016, respectively, while only 49, 152, and 71 genes were upregulated, respectively (Figure 2D). In contrast, normalization based on total read counts showed no significant change in overall mRNA abundance (Figure 2C). Under this method, the number of downregulated genes ranged from 528 to 1,137, while upregulated genes ranged from 1,161 to 1,674 in NSP1-overexpressing cells (Figure 2D). Interestingly, many genes identified as downregulated using ERCC-based normalization were not detected using reads-based normalization, while a larger number of genes appeared falsely upregulated in the reads-normalized data (Figure 2E). A similar pattern was observed when comparing Flag-NSP1 with Flag-NSP1-KH transfection (Figure S1F).

To assess which method more accurately reflected biological changes, we selected a subset of genes exhibiting either high or low expression in Flag-transfected controls for RT-qPCR validation (Figure 2F and Table S1). Most genes that appeared upregulated based on total reads normalization were actually downregulated, consistent with the ERCC normalized results (Figures 2F and 2G). Together, these findings highlight the critical importance of external controls, such as ERCC spike-in controls, when analyzing transcriptomic data in systems with widespread mRNA degradation or translational suppression, such as NSP1-overexpressing cells.

Table S1. Primers design for RT-qPCR, related to Figure 2

### NSP1 repressed the host cell mRNA abundance

**Figure 3:**
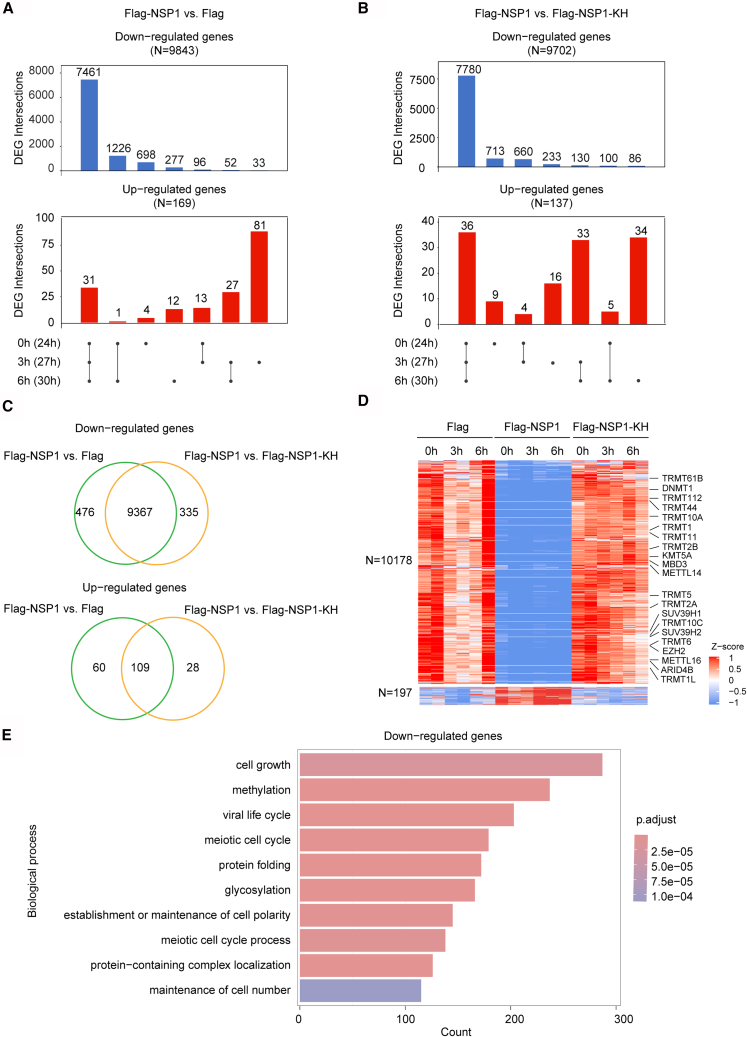
NSP1 suppresses host mRNA abundance (A and B) UpSetR plots showing downregulated (top) and upregulated (bottom) DEGs unique to or shared among 0-, 3-, and 6-h 4sU labeling samples in Flag-NSP1 vs. Flag (A) or Flag-NSP1 vs. Flag-NSP1-KH (B). (C) Venn diagrams showing overlap of DEGs between Flag-NSP1 vs. Flag and Flag-NSP1 vs. Flag-NSP1-KH. (D) Heatmap showing relative expression of combined DEGs from (C). Colors represent Z-score-normalized expression. Some methylation-related genes were labeled. (E) Top 10 enriched GO biological processes for downregulated genes in NSP1-overexpressing cells.

To further study the impact of NSP1 on host gene expression, we analyzed the DEGs identified from normalization using ERCC spike-in controls. For the cells with 4sU labeling 0, 3, and 6 h, a total of 9,843 significantly downregulated genes were identified in NSP1-overexpressed cells compared to Flag-transfected cells. Among these, 7,461 genes (75.8%) were consistently downregulated at all three time points (Figure 3A). Similarly, 9,702 downregulated genes were identified in Flag-NSP1-transfected cells when compared to Flag-NSP1-KH-transfected cells (Figure 3B). Moreover, the vast majority of detected DEGs were observed in both the NSP1 vs. Flag and NSP1 vs. NSP1-KH comparisons (Figure 3C). Furthermore, heatmap analysis showed consistent suppression of mRNA levels across the time course, indicating that approximately 50% of protein-coding genes were repressed at the mRNA level after transfection with NSP1 for more than 24 h (Figure 3D).

We then performed gene ontology (GO) enrichment analysis using DEGs from both the NSP1 vs. Flag and NSP1 vs. NSP1-KH comparisons (Figure 3E and Table S2). We found that most genes involved in protein folding (GO:0006457) and glycosylation (GO:0070085) were significantly downregulated in NSP1-expressing cells. SARS-CoV-2 NSP1 is known to inhibit host protein translation and also disrupt major histocompatibility complex class I (MHC-I) antigen presentation through interference with endoplasmic reticulum (ER)-associated protein folding and glycoprotein maturation, thereby allowing viral evasion of cytotoxic T cell-mediated immune surveillance.^11^^,^^21^^,^^27^ The observed repression of these genes suggests that NSP1 likely diminishes the synthesis and functionality of crucial ER-quality control factors by lowering global host mRNA abundance. This reduction results in impaired assembly of the MHC-I complex and decreased surface expression. Such findings highlight a significant pathogenic mechanism contributing to viral persistence and immune suppression during COVID-19.

Table S2. Enriched GO biological process terms of DEGs detected by the level of total RNA, related to Figure 3

It is noteworthy that a total of 237 genes is associated with methylation processes (GO:0032259), including genes involved in DNA/histone modification (e.g., *DNMT1*, *SUV39H1/H2*, *EZH2*, *MBD3*, *ARID4B*, and *KMT5A*) and RNA modification (e.g., *METTL3*, *METTL14*, *METTL16*, and *TRMT* family members) (Table S2). The result suggests that NSP1 may disrupt the host epigenetic landscape.

Among the relatively few upregulated genes, there is no significantly enriched pathway. However, we observed upregulation of *MASP2* (Mannan-binding lectin serine protease 2) and *NFKBIA* (NF-κB inhibitor alpha), which have been implicated in dampening complement activation and mitigating lung injury during SARS-CoV-2 infection.^28^^,^^29^^,^^30^ These changes highlight the complex transcriptional reprogramming mediated by NSP1 and underscore the potential importance of these genes in modulating inflammation and host response during COVID-19.

### NSP1 protein impeded the synthesis of nascent mRNA in host cells

To elucidate the effect of the NSP1 on nascent RNA synthesis in host cells, we integrated 4sU metabolic labeling into our experimental workflow (Figure 2A). SLAM-seq data were analyzed utilizing GRAND-SLUM software, which quantified both total mRNA abundance and the new-to-total RNA ratio (NTR) for each gene. By combining this with ERCC spike-in normalization, this approach enabled absolute quantification of both pre-existing RNA and nascent RNA.

**Figure 4:**
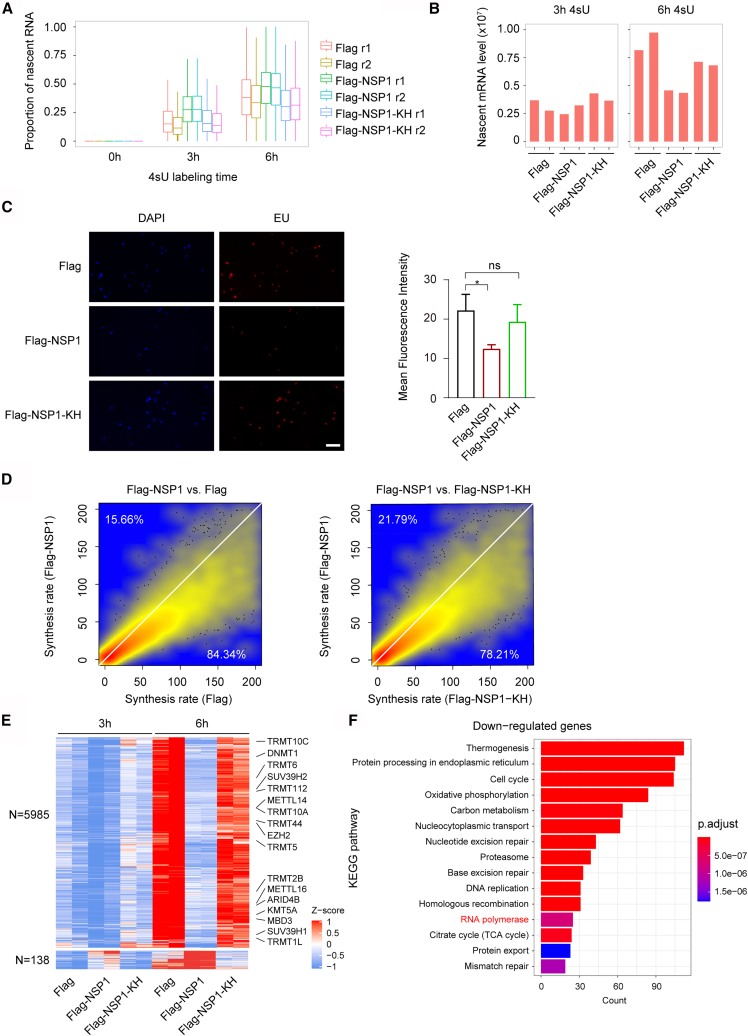
NSP1 inhibits nascent mRNA synthesis (A) Ratio of nascent to total RNA across all 4sU labeling times in cells transfected with the indicated plasmids (r1, r2 = biological replicates). (B) Total nascent RNA levels after 3-h (left) or 6-h (right) 4sU labeling. (C) EU staining of cells expressing Flag, Flag-NSP1, or Flag-NSP1-KH (left). Nuclei stained with DAPI. Scale bars, 100 μm. Mean EU intensity quantified by GraphPad Prism software (right). (*n* = 3 per group; mean ± SD; Student’s *t* test; ∗*p <* 0.05; ns, no significance). (D) Density scatterplots showing lower mRNA synthesis rates in NSP1-expressing cells compared to Flag (left) or NSP1-KH (right). Numbers indicate gene percentages in each cluster. (E) Heatmap showing relative nascent RNA levels of DEGs after 3- or 6-h 4sU labeling. Colors represent Z-score-normalized expression. Some methylation-related genes were labeled. (F) Top 15 enriched KEGG pathways of genes with reduced nascent RNA after NSP1 overexpression.

Following 3- and 6-h 4sU labeling, we observed significant T-to-C conversion rates in the labeled samples, while unlabeled controls (0-h 4sU) showed ≤0.1% conversion, likely attributable to sequencing error (Figure S2A). As expected, conversion rates increased with labeling time and were consistently higher in Flag-NSP1-transfected cells than in Flag or Flag-NSP1-KH controls, indicating accelerated RNA turnover (Figure S2A). Correspondingly, a greater NTR was observed in NSP1-expressing cells, supporting a more dynamic RNA metabolism (Figure 4A), consistent with prior results in SARS-CoV-2-infected Calu-3 cells.^11^

NSP1-overexpressing cells exhibited significant depletion of pre-existing RNA compared to both Flag control and NSP1-KH mutant transfected cells (Figure S2B), confirming that NSP1 is capable of degrading cytosolic mRNAs in host cells.^11^ Notably, we also observed a marked reduction in nascent RNA abundance, particularly after 6 h of 4sU labeling (Figure 4B). To further validate this finding, we employed the uridine analogue 5-ethynyl uridine (EU) incorporation assays to label newly synthesized nuclear RNAs specifically. Fluorescence microscopy showed reduced EU signal in NSP1-expressing cells, indicating suppression of nascent transcript synthesis (Figure 4C).

To quantify transcriptional dynamics, we calculated mRNA half-lives and synthesis rates from the SLAM-seq data. Flag-NSP1-transfected cells exhibited significantly shorter transcript half-lives and reduced synthesis rates compared to both Flag and NSP1-KH controls (Figures S2C and 4D), confirming that NSP1 not only destabilizes existing mRNAs but also strongly inhibits transcriptional output.

To assess the effect of the NSP1 protein on host transcription, we also identified DEGs in the Flag and Flag-NSP1-KH transfected cells according to the nascent RNA abundance. A total of 5,985 downregulated genes and 138 upregulated genes were identified in NSP1 overexpressed cells, compared to Flag or NSP1-KH controls (Figure 4E). Notably, most downregulated nascent DEGs showed concordant changes at the total RNA level (Figure S2D). The functional GO enrichment analysis revealed that the NSP1 protein also suppressed the transcription process of methylation-associated genes, including DNA methyltransferases (*DNMT1*), histone modifiers (*EZH2* and *SUV39H1*), and RNA methyltransferases (*METTLs*, *NSUNs*, and *TRMTs*) (Table S3). The KEGG pathway enrichment results indicated that the NSP1 protein decreased the expression of genes associated with the RNA polymerase pathway, including several members of the *POLR* genes (Figure 4F and Table S4). These findings indicate that NSP1 may interfere with RNA polymerase assembly or stability, thereby coupling translational shutdown with transcriptional silencing.

Table S3. Enriched GO biological process terms of DEGs detected by the level of nascent RNA, related to Figure 4

Table S4. Enriched KEGG pathways of DEGs detected by the level of nascent RNA, related to Figure 4

### NSP1 reduces RNA polymerase II levels and suppresses host transcription

**Figure 5:**
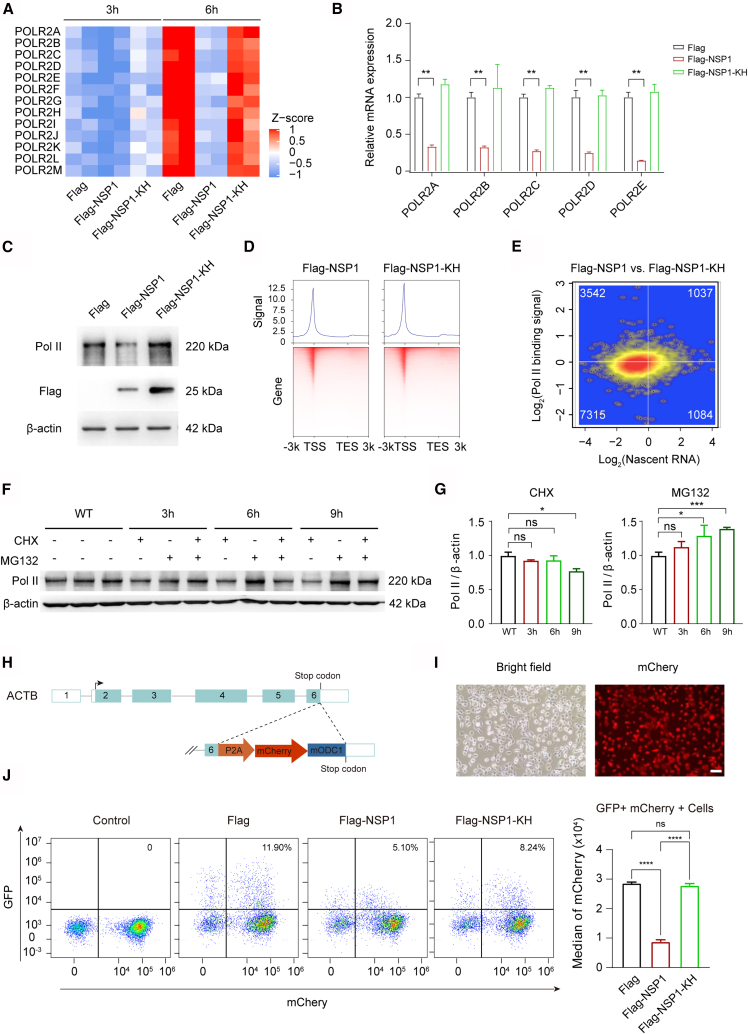
NSP1 decreases RNA polymerase II levels (A) Heatmap showing relative nascent RNA levels of *POLR2* subunits across indicated conditions. (B) RT-qPCR analysis of *POLR2A/B/C/D/E* mRNAs in eGFP-positive cells transfected with the indicated plasmids. Data were normalized to 18S rRNA and presented relative to Flag control (set as 1; *n* = 3 per group; mean ± SD; Student’s *t* test; ∗∗*p* < 0.01). (C) Western blot showing reduced Pol II protein levels in NSP1-expressing cells. β-actin served as a loading control. (D) Average density plots (top) and heatmaps (bottom) of Pol II binding across gene bodies (±3 kb). Colors indicate normalized signal intensities. (E) Scatterplot showing log2-fold changes in Pol II binding across all protein-coding genes. (F) Western blot of Pol II protein levels after treatment with CHX (translation inhibitor) or MG132 (proteasome inhibitor) for 3-, 6-, or 9-h. DMSO served as a negative control; β-actin served as a loading control. (G) Quantification of Pol II/β-actin ratios after CHX (left) or MG132 (right) treatment. Ratios in DMSO-treated controls set as 1 (*n* = 3 per group; mean ± SD; Student’s *t* test; ∗*p <* 0.05; ∗∗∗*p <* 0.001; ns, no significance). (H) Schematic of ACTB-P2A-mCherry-mODC1 cell line with P2A-mCherry-mODC1 cassette inserted before the ACTB stop codon. (I) Brightfield and mCherry fluorescence images of ACTB-P2A-mCherry-mODC1 cells. Scale bars, 100 μm. (J) Flow cytometry analysis of eGFP and mCherry double-positive cells transfected with indicated plasmids (left). Median mCherry fluorescence is shown on the right (*n* = 3 per group; mean ± SD; one-way ANOVA; ∗∗∗∗*p <* 0.0001; ns, no significance).

It is well-established that the transcriptional activity of genes in eukaryotic cells is closely linked to the abundance and functionality of RNA polymerase II (Pol II). We observed that the mRNA transcription activity and expression levels of Pol II subunits were downregulated in NSP1-overexpressing cells (Figures 5A and S2E), which was confirmed by RT-qPCR (Figure 5B). To explore the mechanism underlying the global downregulation of mRNA synthesis observed upon NSP1 overexpression, we assessed the Pol II protein levels in Flag-NSP1-transfected cells. Western blot analysis revealed a significant reduction in Pol II protein abundance in cells expressing NSP1, whereas the mutant NSP1-KH exhibited no such effect relative to Flag-transfected control (Figure 5C). The untagged version of NSP1 recapitulated the inhibition of RNA polymerase II (Figure S2F). We also examined the Pol II binding dynamics in cells overexpressing NSP1 and NSP1-KH protein and found a diminished binding signal in the NSP1-overexpressing cells (Figure 5D). Furthermore, an integrated analysis with SLAM-seq data revealed that approximately 70% (7,315 of the sum of 7,315 and 3,542) genes exhibiting downregulated nascent RNA levels also displayed a corresponding reduction in Pol II binding (Figure 5E). These results indicate that NSP1 contributes to transcriptional repression by downregulating Pol II.

Then, we performed a Co-IP experiment to test whether NSP1 interacts with Pol II, and there was no apparent interaction between NSP1 and Pol II, indicating that NSP1 does not affect the Pol II stability directly (Figures S2G and S2H). To further investigate Pol II turnover dynamics, we employed the protein degradation inhibitor MG132 in conjunction with the protein synthesis inhibitor Cycloheximide (CHX) to test the kinetics of Pol II. In CHX-treated cells, which block new protein synthesis, the Pol II/β-actin ratio progressively declined over 3, 6, and 9 h (Figures 5F and 5G), indicating that Pol II undergoes faster degradation than housekeeping protein. Conversely, in the group administered the protein degradation inhibitor MG132, the Pol II/β-actin ratio increased over time (Figures 5F and 5G), suggesting that the synthesis of Pol II outstripped that of housekeeping protein. Together, these findings indicate that NSP1 does not directly interact with Pol II to mediate degradation, but rather inhibits Pol II transcription and translation through ubiquitination-related proteasome-mediated protein degradation mechanisms, thereby contributing to the widespread transcriptional silencing observed in host cells. This mechanism may represent a key strategy by which SARS-CoV-2 impairs host gene expression to facilitate viral replication.

To further validate the effect of NSP1 on host gene expression, particularly for short half-life transcripts, we developed a live-cell reporter system to monitor ACTB transcription and translation. We inserted a P2A-mCherry-mODC1 cassette immediately upstream of the ACTB stop codon (Figure 5H). The P2A element allows co-translational cleavage, and mCherry is fused to a degradation domain (mouse ODC1) that promotes proteasome-dependent degradation.^31^ Under normal conditions, mCherry fluorescence remains stable, reflecting the balance between ACTB transcription, translation, and degradation (Figure 5I). However, when ACTB expression is inhibited, the production of new mCherry protein drops rapidly, leading to decreased fluorescence.

To assess NSP1’s effect, we transfected those ACTB-ODC1 cells with Flag-NSP1-GFP, Flag-NSP1-KH-GFP (mutant control), or a vector control, and analyzed GFP and mCherry fluorescence by flow cytometry. We observed a reduced mCherry fluorescence in GFP and mCherry double-positive cells in NSP1-expressing cells, compared to cells transfected with vector or NSP1-KH-GFP, suggesting inhibition of ACTB transcription and/or translation (Figure 5J). These findings provide functional evidence that NSP1 reduces host gene expression at both transcriptional and translational levels, and that this suppression preferentially affects labile transcripts or proteins with rapid turnover.

## Discussion

The SARS-CoV-2 NSP1 disrupts cellular gene expression through multiple mechanisms, including the inhibition of translation, mRNA degradation, and the export of nuclear mRNA.^11^^,^^21^ Given that the overall quantities of mRNA have altered in response to viral infection or NSP1 transfection, it is imperative to implement a more robust normalization method to ensure accurate assessments of gene expression, particularly regarding mRNA transcription progress. Utilizing ERCC spike-in RNAs and SLAM-seq technologies, our findings highlight a previously underexplored mechanism by which the NSP1 protein of SARS-CoV-2 modulates host gene expression at the transcriptional level. Consistent with its known role as a suppressor of host translation, NSP1 exerts its effects by promoting the degradation of host mRNAs, effectively silencing the expression of host proteins. Notably, our results suggest that this degradation includes mRNAs encoding RNA polymerase II, resulting in a significant reduction of Pol II protein levels. As Pol II is the central enzyme responsible for the transcription of protein-coding genes, its depletion leads to a global repression of mRNA transcription progress. This dual inhibition, at both the transcriptional and translational levels, reflects a sophisticated strategy by which SARS-CoV-2 reprograms the host cell environment to suppress antiviral responses and favor viral replication. Pol II levels could be regulated by multiple factors, particularly the stress during viral infection.^32^^,^^33^ Our results showed that NSP1 overexpression promotes Pol II degradation through proteasome-dependent pathways (Figures 5H–5J). Although Pol II-mediated transcription is known to be repressed upon viral infection,^32^ the underlying molecular mechanisms remain unclear. Here, we demonstrate that Pol II was much more vulnerable to ubiquitination-related proteasome degradation. NSP1 protein may not directly interact with Pol II to mediate its degradation but rather accelerate Pol II depletion and contribute to the widespread transcriptional silencing in host cells.

Normalization constitutes a fundamental yet usually overlooked component of transcriptome data analysis. If the cellular sources produce equivalent quantities of RNA per cell, and the yields of RNA and its derivatives remain consistent throughout the experimental manipulation, then normalized expression data should provide an accurate representation of the relative levels of each gene product. However, numerous studies have demonstrated that the NSP1 protein of SARS-CoV-2 possesses the ability to broadly induce cellular mRNA degradation and inhibit host protein translation,^9^^,^^10^^,^^11^^,^^12^^,^^13^^,^^14^^,^^15^^,^^16^^,^^17^ and similar results have also been found in this study (Figure 1D). In this context, traditional normalization methodologies that do not incorporate standardized controls may result in inaccurate interpretations concerning mRNA abundance (Figure 2C) and subsequently affect the identification of NSP1 protein-induced genes and their corresponding biological functions (Figures 2D, 2E, and 3E). In contrast, through the normalization process utilizing ERCC spike-in controls, it is observed that the NSP1 protein significantly inhibits the mRNA abundance of each gene within host cells (Figures 2C–2G and 3E). Our study provides a more accurate assessment for exploring the influences of the NSP1 protein on the host cells.

Despite numerous studies, it remains unclear how the NSP1 protein regulates mRNA transcription. Our study utilized 4sU labeling technology to directly assess the absolute quantities of nascent RNA for each gene and further evaluated the function of NSP1 protein on transcription progress. It has been determined that the NSP1 protein has the potential to inhibit the synthesis of nascent RNA in more than 5,000 genes (Figure 4E). These genes were enriched in the function of methylation and viral life cycle, indicating that the NSP1 protein disrupts the transcription process of methylation-associated genes and subsequently evades immune detection (Figure 4F). Mechanistically, this extensive influence is achieved through the degradation of RNA polymerase II protein (Figure 5). This suppression of Pol II-dependent transcription has broad implications for host defense. Many innate immune genes, including interferons and proinflammatory cytokines, rely on Pol II-mediated transcription for their expression. By inhibiting this pathway, NSP1 effectively blunts the host’s ability to mount an early immune response. This is consistent with the observed delay in interferon responses in COVID-19 patients and contributes to the ability of the virus to establish infection before immune clearance mechanisms are activated.^34^ Therefore, NSP1 represents a critical node in the host-virus interaction that promotes viral pathogenicity by disabling host transcriptional machinery.

These findings underscore significant opportunities for therapeutic intervention. One potential approach is to directly target NSP1, utilizing small-molecule inhibitors that disrupt its interaction with the ribosome or its RNA degradation machinery. Such a strategy could help restore host gene expression and enhance innate immunity. Additionally, stabilizing RNA Pol II or increasing its expression during infection may counteract NSP1’s inhibitory effects, thereby preserving essential transcriptional activity in the early stages of infection. Moreover, since NSP1 is conserved across various coronaviruses,^35^ these strategies could be effective not only for COVID-19 but also for addressing future emerging coronavirus infections.

Beyond antiviral therapy, the molecular behavior of NSP1 presents unique opportunities for molecular biology research. Its potent and specific impact on mRNA stability and transcription dynamics provides a valuable tool for probing mRNA metabolism and transcriptional regulation. For instance, NSP1 could be employed in experimental systems to investigate the dynamics of transcription shutdown and recovery, or to study the half-life and decay kinetics of different mRNA species under controlled suppression. These applications could yield insights into the regulation of gene expression under stress or pathogenic conditions.

In conclusion, our work expands the understanding of NSP1’s role beyond translation inhibition to include a significant influence on transcriptional regulation. By reducing Pol II levels, NSP1 orchestrates a broad shutdown of host gene expression, reinforcing its role as a key factor in immune evasion and viral pathogenicity. Targeting NSP1 function, or the pathways it disrupts, holds promise for therapeutic development and offers a novel tool for dissecting RNA biology in mammalian cells.

### Limitations of the study

Our study is not without limitations. One important consideration is the context of natural viral infection. In our experimental models, NSP1 expression was introduced in isolation or at controlled levels. In an actual infection scenario, NSP1 is expressed alongside multiple other viral proteins, some of which may modulate or counterbalance its effects. Moreover, various viral proteins may induce a rapid activation of interferon-stimulated genes (ISGs) prior to the onset of repression mediated by the NSP1 protein. Additionally, the abundance and timing of NSP1 expression during the viral replication cycle may vary across cell types and host conditions. As such, the extent to which NSP1 contributes to transcriptional repression during actual SARS-CoV-2 infection remains to be fully defined. Future studies using live virus under biosafety level 3 (BSL-3) conditions, coupled with single-cell transcriptomics, would be valuable to precisely measure NSP1 activity and its contribution to global host transcriptional changes.

## Resource availability

### Lead contact

Further information and requests for resources and data should be directed to and will be fulfilled by the lead contact, Zheng Li (li_zheng@gzlab.ac.cn).

### Materials availability

All unique/stable reagents generated in this study are available from the lead contact with a completed materials transfer agreement.

### Data and code availability

• The raw sequence data of SLAM-seq and CUT&Tag Seq have been deposited in the National Genomics Data Center (NGDC), China National Center for Bioinformation (CNCB)/Beijing Institute of Genomics, Chinese Academy of Sciences (GSA: HRA012036) that are publicly accessible at https://ngdc.cncb.ac.cn/gsa.
• This paper does not report original code.
• Any additional information required to reanalyze the data reported in this paper is available from the lead contact upon request.

## Acknowledgments

This work was supported by the Young Scientists Program of Guangzhou Laboratory, grant no. QNPG24-08 (to J.L.) and Science and Technology Projects in Guangzhou, grant no. 2023A04J0166 (to J.L.). Z.L. was supported by Major Project of Guangzhou National Laboratory (GZNL2023A02003 and GZNL2025C02025) and the 10.13039/501100001809National Natural Science Foundation of China (NSFC 32170596). H.P. was supported by the Fund of Shenzhen Key Laboratory (ZDSYS20220606100803007). We thank Dr. Dong Wang and Dr. Ting Huang for assisting with experiments and providing advice on virus infection procedures.

## Author contributions

Z.L. conceived the project; J.L. performed the computational analysis and interpreted the data; K.W. performed the majority of experiments and data collection, with technical assistance from S.W.; J.W. performed the ACTB-ODC1 experiments and collected data; C.Z. assisted in data interpretation; J.L., K.W., and Z.L. wrote the manuscript, with H.P. offering valuable suggestions. All authors read and approved the final manuscript.

## Declaration of interests

The authors declare no competing interests.

## STAR★Methods

### Key resources table

**Table:** 

REAGENT or RESOURCE	SOURCE	IDENTIFIER
**Antibodies**		

β-actin	CST	Cat#4970, RRID: AB_2223172
GADPH	CST	Cat#2118, RRID: AB_561053
GFP	Abcam	Cat#ab290, RRID: AB_303395
mCherry	Abcam	Cat#ab183628, RRID: AB_2650480
NSP1	Abcam	Cat#ab284631, RRID: AB_3717487
Lamin A/C	CST	Cat#4777, RRID: AB_10545756
Pol II	Sigma Aldrich	Cat#05-623, RRID: AB_309852
Flag	CST	Cat#14793, RRID: AB_2572291
Rabbit IgG	CST	Cat#66362, RRID: AB_2924329
Donkey anti-Mouse IgG (H + L) Highly Cross-Adsorbed Secondary Antibody, Alexa Fluor™ Plus 488	Thermo Fisher Scientific	Cat#A32766, RRID: AB_2762823
Donkey anti-Rabbit IgG (H + L) Highly Cross-Adsorbed Secondary Antibody, Alexa Fluor™ Plus 594	Thermo Fisher Scientific	Cat#A32754, RRID: AB_2762827
Anti-mouse IgG HRP-linked Antibody	Cell Signaling Technology	Cat#7076, RRID: AB_330924
Anti-rabbit IgG HRP-linked Antibody	Cell Signaling Technology	Cat#7074, RRID: AB_2099233

**Bacterial and virus strains**		

DH5α	TSINGKE	Cat#TSC-C01

**Chemicals, peptides, and recombinant proteins**		

DAPI	Sigma Aldrich	Cat#D9542
Mounting medium	Invitrogen	Cat#P36982
RPMI1640	Gibco	Cat#C11875500BT
DMEM	Gibco	Cat#C11965500BT
Fetal Bovine Serum	Capricorn Scientific	Cat#FBS-12A
DPBS	Thermo Fisher Scientific	Cat#C20012500BT
Penicillin Streptomycin	Gibco	Cat#15140122
Trypsin	Thermo Fisher Scientific	Cat#25300054
Lipofectamine 3000	Invitrogen	Cat#L3000015
MG132	MCE	Cat#HY-13259
Cycloheximide	MCE	Cat#HY-12320
4sU	Sigma Aldrich	Cat#T4509
Iodoacetamide	Sigma Aldrich	Cat#A3221
EtOH	Sangon	Cat#A500737
Glycogen	Roche	Cat#10901393001
HiScript III RT SuperMix	Vazyme	Cat#R323
Taq Pro Universal SYBR qPCR Master Mix	Vazyme	Cat#Q712
loading buffer	Monad	Cat#PE40101S
Protease Inhibitor Cocktail	Beyotime	Cat#P1045
External RNA Control Consortium (ERCC)	Thermo Fisher Scientific	Cat#4456740
Paraformaldehyde	Biosharp	Cat#BL539A
Trizol	Invitrogen	Cat#15596018
Ampicillin	Sangon	Cat#A610028
SurePAGE™ Midi, Bis-Tris, 4-12% (SDS-PAGE gel)	Genscript	Cat#M00995
Agarose	Sangon	Cat#A620014
Ultra GelRed	Vazyme	Cat#GR501
Methanol	Sangon	Cat#A506806
Agar	Sangon	Cat#A505255
Tryptone	Sangon	Cat#A650217
Yeast Extract	Sangon	Cat#A610961
Glycine	Sangon	Cat#A610235
Pierce Protein A/G Magnetic Beads	Thermo Fisher Scientific	Cat#88802
ECL Western Blotting Substrate	Tanon	Cat#180-5001
Gelatin	Sigma Aldrich	Cat#G9391
Immobilon-P PVDF Membrane	Millipore	Cat#IPVH00010
Normal Donkey Serum	Yeasen	Cat#36116ES10
Bovine Serum Albumin	Sigma Aldrich	Cat#A1933
Triton X-100	Sigma Aldrich	Cat#X100

**Critical commercial assays**		

VAHTS Universal V6 RNA-seq Library Prep Kit	Vazyme	Cat#NR604
BeyoClick™ EU-594 RNA Synthesis Assay Kit	Beyotime	Cat#R0309S
Vazyme Hyperactive® Universal CUT&Tag Assay Kit for Illumina	Vazyme	Cat#TD904
Immunoprecipitation Kit with Protein A+G Magnetic Beads	Beyotime	Cat#P2179S

**Deposited data**		

Raw sequence data of SLAM-seq and CUT&Tag Seq	This paper	GSA: HRA012036

**Experimental models: Cell lines**		

NCI-H1299	Cell Bank of Chinese Academy of Sciences	Cat#SCSP-589, RRID:CVCL_0060
HEK-293T	Cell Bank of Chinese Academy of Sciences	Cat#SCSP-502, RRID:CVCL_0063

**Oligonucleotides**		

Primer sequences. See Table S1	This paper	N/A

**Recombinant DNA**		

pcDNA4-Flag (Flag)	This paper	N/A
pcDNA4-Flag-NSP1 (Flag-NSP1)	This paper	N/A
pcDNA4-Flag-NSP1-KH (Flag-NSP1-KH)	This paper	N/A
pcDNA4-NSP1 (untagged-NSP1)	This paper	N/A
pcDNA4-NSP1-KH (untagged-NSP1-KH)	This paper	N/A
pcDNA4-mCherry	This paper	N/A
pcDNA4-eGFP	This paper	N/A

**Software and algorithms**		

GraphPad Prism	GraphPad Software	RRID: SCR_002798
ImageJ	NIH	RRID:SCR_003070
STAR v2.7.2b	Dobin et al. (2013)	https://github.com/alexdobin/STAR/releases/tag/2.7.2b
GRAND-SLAM	Jürges et al. (2018)	https://github.com/erhard-lab/gedi/wiki/GRAND-SLAM
DESeq2 v1.26.0	Love et al. (2014)	N/A
UpSetR v1.4.0	Conway et al. (2017)	N/A
Bowtie2	Langmead et al. (2012)	N/A
MACS2	Zhang et al. (2008)	N/A
BEDTools	Quinlan et al. (2010)	N/A
deepTools	Ramírez et al. (2014)	N/A

### Experimental model and study participant details

#### Cell lines

The H1299 cell line was obtained from the Cell Bank of Chinese Academy of Sciences (SCSP-589) and maintained in RPMI1640 (C11875500BT, Gibco) with 10% Fetal Bovine Serum (FBS-12A, Capricorn Scientific) and 1% Penicillin Streptomycin (15140122, Gibco). The HEK-293T cell line was obtained from the Cell Bank of Chinese Academy of Sciences (SCSP-502) and maintained in DMEM (C11965500BT, Gibco) with 10% Fetal Bovine Serum and 1% Penicillin Streptomycin. Cells were cultured at 37 °C under 5% CO_2_. All cell lines used in this study were routinely tested and confirmed to be free of mycoplasma contamination by PCR.

### Method details

#### Plasmid construction

All expression plasmids were generated using the pcDNA4-Flag vector backbone. The NSP1 coding sequence was amplified from a donor plasmid, while the NSP1-KH (K164A/H165A) mutant was generated via site-directed mutagenesis based on NSP1 sequence. Subsequently, the NSP1, NSP1-KH, mCherry and eGFP fragments were cloned into pcDNA4 vector by seamless cloning (C115, Vazyme). The leader sequence (ATTAAAGGTTTATACCTTCCCAGGTAACA-AACCAACCAACTTTCGATCTCTTGTAGATCTGTTCTCTAAACGAAC) was synthesized and fused to the 5′-terminus of either NSP1 or NSP1-KH sequences via seamless cloning.^20^ To enable fluorescence-activated cell sorting (FACS), an IRES-eGFP cassette was cloned downstream of the NSP1 or NSP1-KH coding sequence to facilitate their co-expression (Data S1). To generate the untagged version of NSP1 and NSP1-KH plasmids, the Flag tag sequence was removed using site-directed mutagenesis PCR.

#### Cell transfection

For the plasmid transfection assay, cells were seeded in a six-well plate and cultured to 60-70% confluence. Transfection was performed using Lipofectamine 3000 (L3000015, Invitrogen) according to the manufacturer’s protocol. A total of 2 μg plasmid DNA was transfected per well. In groups receiving less than 2 μg of specific plasmid(s), an empty vector plasmid was added to equalize the total DNA amount across all conditions. After transfection, cells were incubated for 24 h prior to downstream analysis.

#### Protein stability assay

H1299 cells were cultured to 60-70% confluence in six-well plates and treated with either MG132 (10 μM, HY-13259, MCE), Cycloheximide (CHX) (100 μg/mL, HY-12320, MCE), or a combination of MG132 and CHX for 3, 6, and 9 hours. Following treatment, cells were detached using 0.05% Trypsin (25300054, Thermo Fisher Scientific) and resuspended in DPBS and collected. Equivalent cell numbers from each condition were lysed and processed for Western blot analysis to assess protein stability.

#### Flow cytometry

Twenty-four hours post-transfection, cells were harvested by trypsinization, washed twice with cold DPBS, and resuspended in staining buffer (DPBS supplemented with 2% FBS). Cell suspensions were filtered through a 40 μm nylon mesh to remove clumps. eGFP-positive cells were sorted on a Sony MA900 cell sorter (Sony) using the FITC channel (excitation at 488 nm, emission at 530/30 nm). Live single cells were gated based on forward and side scatter parameters. Un-transfected cells were used to establish the gating threshold for eGFP-negative populations. Sorted eGFP-positive cells were collected into tubes containing culture medium supplemented with 10% FBS and used for RNA extraction and downstream analysis.

#### 4sU labeling for SLAM-seq

For metabolic RNA labeling, H1299 cells were transfected for 24 hours and subsequently incubated in fresh medium containing 500 μM 4sU (T4509, Sigma) for 0 hours (No 4sU control), 3 hours, and 6 hours. Following labeling, eGFP-positive cells were isolated by FACS, and the total RNA was extracted using Trizol reagent. The External RNA Control Consortium (ERCC, 4456740, Thermo Fisher Scientific) spike-in controls were added to the same amount of RNA from cells under different transfections in each sample according to the manufacturer’s instructions. The mixture of total RNA was subsequently treated with 10 mM Iodoacetamide (A3221, Sigma) (+IAA) or EtOH (A500737, Sangon) (−IAA) in standard reaction conditions. Subsequently, polyA-tailed RNA was selected using Dynabeads oligo (dT) (Thermo Fisher Scientific). Standard RNA-Seq libraries were prepared using the VAHTS Universal V6 RNA-seq Library Prep Kit for Illumina (NR604, Vazyme) and deeply sequenced on an Illumina NovaSeq X Plus system (150 cycles, paired-end).

#### SLAM-seq data analysis and ERCC spike-in normalization

Alignment of SLAM-seq reads was performed using STAR v2.7.2b.^36^ Initially, reads were aligned to a combined reference of human rRNA and tRNA, and all aligned reads were filtered out. The remaining reads were aligned to a reference containing the human genome (ENSEMBL GRCh38), the full length of plasmid vectors, and the 92 ERCC spike-in controls using STAR with parameters ‘--outFilterMismatchNmax 10 --outFilterMultimapNmax 1 --outSAMattributes MD NH’. All bam files for each sample were merged and converted into a CIT file using the GEDI toolkit and then processed using GRAND-SLAM with parameters trimming 13 nucleotides from the 5′ ends and 5 nucleotides from the 3′ ends of each read.^37^ The output of GRAND-SLAM includes the raw counts of total RNA and nascent RNA for each gene, as well as the estimated ratio of newly synthesized molecules to total molecules for each gene (new to total ratio, NTR). The raw read counts of ERCC spike-in controls were utilized to calculate normalization factors for each sample, including both transfected and un-transfected samples. These normalization factors were employed to scale the levels of total RNA and nascent RNA for each gene. DESeq2 v1.26.0^38^ was used to identify significantly differentially expressed genes (DEGs) in Flag-NSP1-transfected samples compared with Flag- and Flag-NSP1-KH-transfected samples at 4sU labeling 0, 3, and 6 hours, independently (|fold change| ≥ 2; False Discovery Rate (FDR) < 0.05). Visualization of intersecting DEGs was plotted using UpSetR v1.4.0.^39^ The ‘ClusterProfilter’ package in R was employed for the functional enrichment analysis of DEGs in gene ontology (GO) and KEGG pathways.^40^ The half-life of each gene within the respective samples was evaluated through linear regression analysis of the logarithmic values of the calculated old transcript fraction. The estimated variances derived from the GRAND-SLAM methodology were employed as weights in this regression analysis. The regression coefficient, denoted as *lambda* (λ), was subsequently converted to half-life using the formula: -log(2)/λ.

#### Real-time quantitative PCR

Total RNA was isolated from H1299 using Trizol (15596018, Invitrogen), and the single-strand cDNA was synthesized using HiScript III RT SuperMix (R323, Vazyme). Real-time PCR was performed on CFX-96 (BIO-RAD) with the Taq Pro Universal SYBR qPCR Master Mix (Q712, Vazyme). Gene-specific primers were designed using the NCBI primer-blast. All values were normalized to the abundance of endogenous 18S rRNA. The PCR amplification protocol involved an initial denaturation step at 95 °C for 5 minutes, followed by 40 cycles of PCR at 95 °C for 15 s, 60 °C for 30 s. Data were presented as the average of triplicate experiments ± standard deviation (SD).

#### Western blot

Cells were lysed directly in 1.25 × loading buffer (PE40101S, Monad) supplemented with Protease Inhibitor Cocktail (P1045, Beyotime). Lysates were loaded onto 6%-12% SDS-PAGE gels and, followed by electrophoresis and transferred to a 0.45 μm PVDF membrane (IPVH00010, Millipore). After blocking the membranes with 5% non-fat dried milk in Tris buffered saline (TBS) supplemented with 0.1% Tween-20 at room temperature for 1 hour, they were incubated overnight at 4 °C with primary antibodies. The membranes were washed with TBST for 10 minutes 3 times and then incubated with the secondary antibodies at room temperature for 1 hour. Following a 30-minute wash with TBST, chemiluminescence measurements were performed using substrate (180-5001, Tanon).

#### Immunofluorescence staining

Following transfection, H1299 cells were fixed in 4% paraformaldehyde (PFA) (BL539A, Biosharp) for 20 minutes at room temperature, washed three times with DPBS, and permeabilized with 0.1% Triton X-100 for 5 minutes at room temperature. After blocking with 5% donkey serum for 30 minutes at room temperature, slides were incubated with primary antibodies at 4 °C overnight and then washed three times with DPBS and incubated with fluorescent-conjugated secondary antibodies (1:200-1:400) at room temperature for 1 hour. The slides were incubated in DPBS containing 1 μg/mL DAPI (D9542, Sigma) for 10 minutes at room temperature. Following three additional washes with DPBS, slides were mounted with Mounting medium (P36982, Invitrogen) and photographed. Images were acquired using ZEISS Axioscope 5 and ZEISS LSM 900 confocal microscope and analyzed with ImageJ software.

#### Antibodies

We used following antibodies for Western blot, immunoprecipitation and immunofluorescence staining: β-actin (4970, CST), GADPH (2118, CST), GFP (ab290, Abcam), mCherry (ab183628, Abcam), NSP1 (ab284631, Abcam), Lamin A/C (4777, CST), Pol II (05-623, Sigma Aldrich), Flag (14793, CST), and Rabbit IgG (66362, CST).

#### Immunoprecipitation

Following transfection, H1299 cells were harvested, washed once with ice-cold PBS, and lysed on ice for 30 min at a density of 1 × 10^6^ cells per 200 μL lysis buffer supplemented with protease and phosphatase inhibitors (P2179S, Beyotime). Lysates were clarified by centrifugation at 14,000 × g for 10 min at 4 °C. Supernatants were incubated overnight at 4 °C with anti-Flag, anti-Pol II, rabbit IgG (66362, CST) and mouse IgG (P2179S, Beyotime). Ten percent of each lysate was retained as input control. Antigen-antibody complexes were captured by adding 20 μL of pre-washed Pierce Protein A/G Magnetic Beads (88802, Thermo Scientific) and incubating for 2 hours at 4 °C. Beads were washed 3-4 times with lysis buffer, and bound complexes were eluted in SDS loading buffer by boiling at 95 °C for 10 min. Samples were analyzed by Western blot to detect protein interaction.

#### EU staining

Based on the instructions of the BeyoClick™ EU-594 RNA Synthesis Assay Kit (R0309S, Beyotime), the detection of newly synthesized RNA in H1299 cells was performed as follows. H1299 cells transfected with Flag, Flag-NSP1, or Flag-NSP1-KH plasmids were first sorted and seeded. After a 12-hour culture period on glass slides, the cells were then incubated with 1 mM EU for 2 hours at 37 °C. After labeling, cells were fixed with 4% paraformaldehyde for 15 minutes at room temperature, followed by permeabilization with 0.3% Triton X-100 in PBS for 15 minutes. The click chemistry reaction was carried out by incubating the cells with a freshly prepared reaction mixture containing Azide 594 and CuSO_4_ for 30 minutes at room temperature, protected from light. Following the reaction, cells were washed thoroughly with the provided wash buffer. Nuclei were counterstained with Hoechst 33342, and fluorescent signals were visualized using ZEISS Axioscope 5 microscope.

#### Pol II CUT&Tag Seq library construction

Pol II CUT&Tag Seq libraries were performed using the Vazyme Hyperactive® Universal CUT&Tag Assay Kit for Illumina (TD904) following the manufacturer’s protocol. eGFP-positive H1299 cells were FACS-sorted from Flag-NSP1-, and Flag-NSP1-KH-transfected cells. Each sample (30,000 cells) was washed with wash buffer, and nuclei were isolated in NE buffer for 10 min at 4 °C. Nuclei were bound to activated Concanavalin A-coated magnetic beads and incubated overnight at 4 °C with 0.5 μg primary antibody (anti-Pol II, Sigma Aldrich, 05-623). After removal of unbound antibody, nuclei were incubated with goat anti-mouse IgG for 1 hour at room temperature, followed by incubation with pA/G-Tn5 transposase for 1 hour at room temperature. Chromatin was fragmented using TTBL buffer. After an equal amount of spike-in DNA was added to each sample, DNA fragments were purified with DNA extraction beads. Libraries were amplified using universal i5 and uniquely barcoded i7 primers, with different barcodes for each sample, and purified using DNA clean beads (Vazyme, N411). Libraries were deeply sequenced on an Illumina NovaSeq X Plus system (150 cycles, paired-end).

#### Pol II CUT&Tag Seq data analysis with spike-in normalization

Raw sequencing reads were processed using TrimGalore v0.5.0 (--quality 20 --length 30 --nextera) to remove adaptors and low-quality bases. Retained reads were aligned to a reference containing the human genome (ENSEMBL GRCh38), the full length of plasmid vectors, and the CUT&Tag spike-in DNA using Bowtie2 with the --very-sensitive-local option.^41^ For those reads aligned to the human genome, the mitochondrial reads and PCR duplicates were removed, and only uniquely mapped reads were retained for downstream analysis. Peak calling was performed using MACS2 (-q 0.05) to identify regions enriched in Pol II binding.^42^ BEDTools was used to merge high-confidence peaks across replicates to generate consensus binding regions.^43^ Raw read counts within these regions, as well as the promoter regions of protein-coding genes, were counted by the deepTools module multiBamSummary.^44^ The reads aligned to the CUT&Tag spike-in DNA reference were quantified to derive normalization factors for each sample. These factors were subsequently employed to compute the Pol II binding signal across the entire human genome. In parallel, BAM files were converted to normalized BigWig format using the deepTools module bamCoverage with designated “--scaleFactor” values for each sample.^44^

### Quantification and statistical analysis

The data were subjected to statistical analysis using GraphPad Prism software and were presented as the mean ± standard deviation (SD). All statistical analyses were described in the figure legends. Statistical significance was determined at the probability threshold of 0.05 (*p <* 0.05): ∗ *p <* 0.05, ∗∗ *p <* 0.01, ∗∗∗ *p <* 0.001, and ∗∗∗∗ *p <* 0.0001. The values represented biological replicates, and all the experiments were conducted with a minimum of three replicates.

## Supplemental information

Document S1. Figures S1 and S2

Data S1. The sequences of constructed plasmids

Data S2. The raw data of western blots
