# Supplementary material for: SARS-CoV-2 nonstructural protein 1 suppresses host transcription by reducing RNA polymerase II levels
Source: iScience. 2025 Nov 26;28(12):114233. doi: 10.1016/j.isci.2025.114233 (PMC12741407; doi:10.1016/j.isci.2025.114233)
Supplement: Document S1. Figures S1 and S2 [file mmc1.pdf]

iScience, Volume *28*

Supplemental information

SARS-CoV-2 nonstructural protein 1 suppresses host transcription by reducing RNA polymerase II levels

Jianfang Li, Kang Wang, Jie Wang, Caizhou Zhong, Shenglan Wang, Hong Peng, and Zheng Li

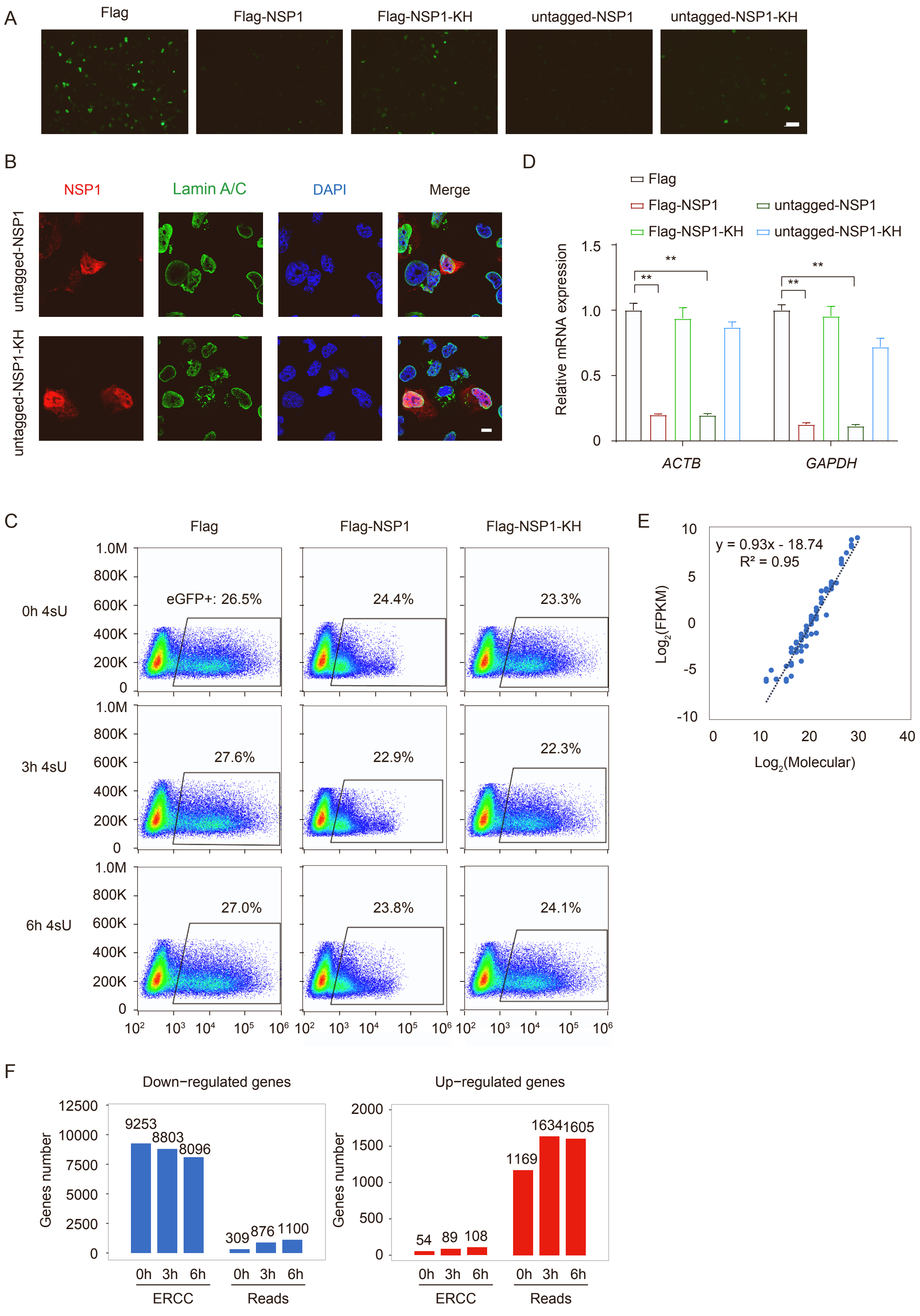
A
Flag
Flag-NSP1
Flag-NSP1-KH
untagged-NSP1
untagged-NSP1-KH
B
NSP1
Lamin A/C
DAPI
Merge
untagged-NSP1
untagged-NSP1-KH
D
Flag
Flag-NSP1
Flag-NSP1-KH
untagged-NSP1
untagged-NSP1-KH
Relative mRNA expression
**
ACTB
GAPDH
C
Flag
Flag-NSP1
Flag-NSP1-KH
0h 4sU
3h 4sU
6h 4sU
eGFP+: 26.5%
24.4%
23.3%
27.6%
22.9%
22.3%
27.0%
23.8%
24.1%
E
y = 0.93x - 18.74
R² = 0.95
Log2(FPKM)
Log2(Molecular)
F
Down−regulated genes
Up−regulated genes
Genes number
9253
8803
8096
309
876
1100
54
89
108
1169
1634
1605
0h 3h 6h
ERCC
Reads

Figure S1. The effect of NSP1 on host cells (related to Figures 1 and 2). (A) eGFP levels in H1299 cells expressing the indicated plasmids. Scale bar, 100 μm. (B) Immunofluorescence analysis of H1299 cells transfected with untagged NSP1 or NSP1-KH plasmids. NSP1 proteins were detected using anti-NSP1 antibody (red), the nuclear envelope marker Lamin A/C (green), and DAPI (blue). Scale bar, 10 μm. (C) Flow cytometry sorting of eGFP-positive cells for SLAM-seq analysis. Numbers indicate eGFP+ percentages (n = 2 per group). (D) RT-qPCR analysis of *ACTB* and *GAPDH* mRNA levels in eGFP-positive cells. Data normalized to 18S rRNA and presented relative to Flag control (set as 1; n = 3 per group; mean ± SD; Student's t-test; ** $p < 0.01$). (E) Correlation between ERCC spike-in input and FPKM values. (F) Number of DEGs detected using ERCC or reads normalization (Flag-NSP1 vs. Flag-NSP1-KH) after 0-, 3-, or 6-hour 4sU labeling.

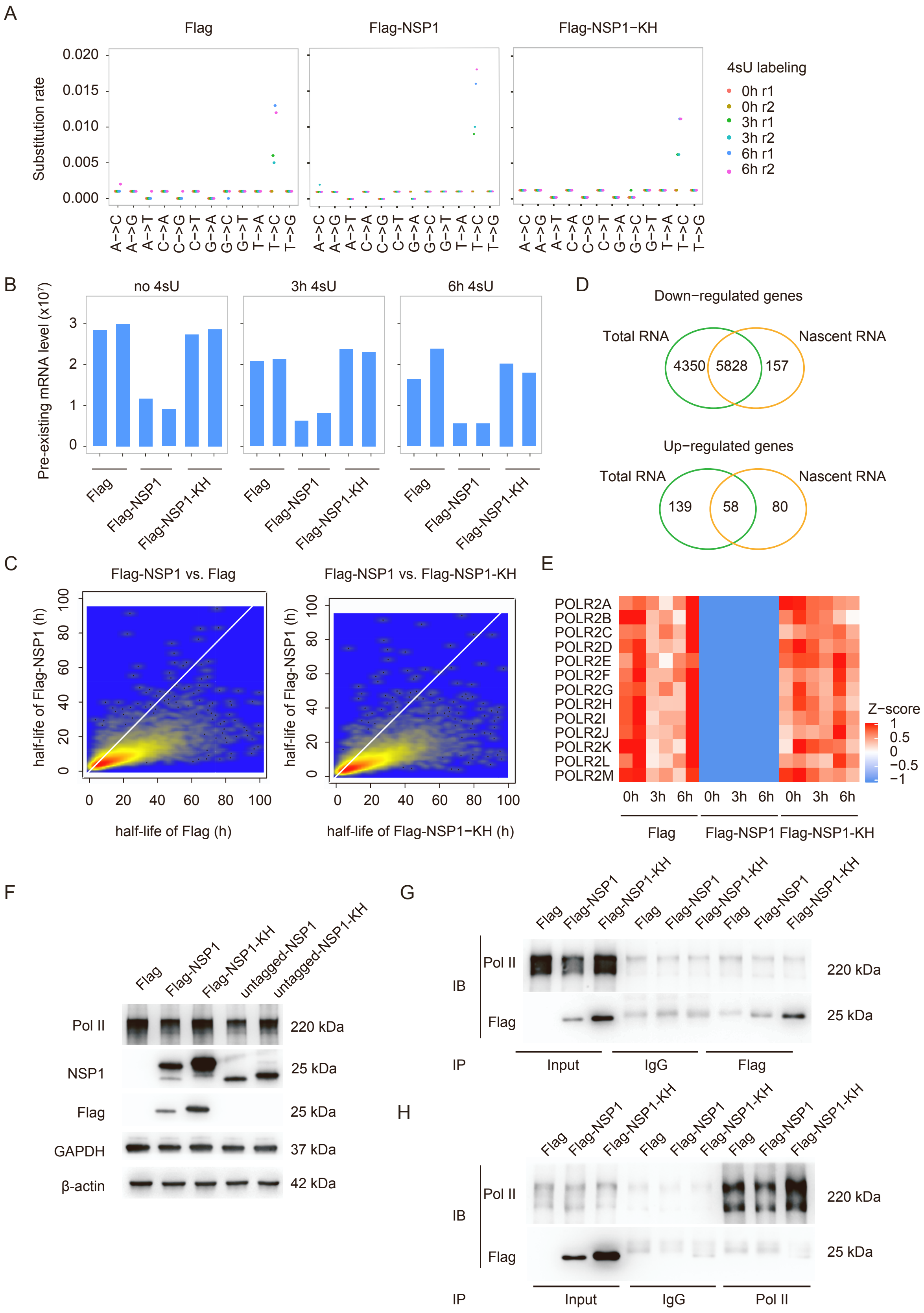

A
Flag
Flag-NSP1
Flag-NSP1-KH
Substitution rate
0.020
0.015
0.010
0.005
0.000
A->C A->G A->T C->A C->G C->T G->A G->C G->T T->A T->C T->G
4sU labeling
0h r1
0h r2
3h r1
3h r2
6h r1
6h r2
B
no 4sU
3h 4sU
6h 4sU
Pre-existing mRNA level (x10^7)
Flag
Flag-NSP1
Flag-NSP1-KH
D
Down-regulated genes
Total RNA
Nascent RNA
4350
5828
157
Up-regulated genes
139
58
80
C
Flag-NSP1 vs. Flag
Flag-NSP1 vs. Flag-NSP1-KH
half-life of Flag-NSP1 (h)
half-life of Flag (h)
half-life of Flag-NSP1-KH (h)
E
POLR2A
POLR2B
POLR2C
POLR2D
POLR2E
POLR2F
POLR2G
POLR2H
POLR2I
POLR2J
POLR2K
POLR2L
POLR2M
Z-score
0h 3h 6h
Flag
Flag-NSP1
Flag-NSP1-KH
F
untagged-NSP1
untagged-NSP1-KH
Pol II
NSP1
GAPDH
β-actin
220 kDa
25 kDa
37 kDa
42 kDa
G
IB
IP
Input
IgG
H

Figure S2. NSP1 destabilizes host existing mRNAs (related to Figures 4 and 5). (A) Nucleotide substitution rates showing efficient 4sU-to-C conversion in labeled vs. unlabeled cells (r1, r2 = biological replicates). (B) Total pre-existing mRNA levels across samples at 0-, 3-, and 6- hr 4sU labeling. (C) Scatterplots of mRNA half-lives in Flag-NSP1 vs. Flag (left) or Flag-NSP1 vs. Flag-NSP1-KH (right) cells after 6-hour labeling. (D) Venn diagrams of DEGs identified from total RNA vs. nascent RNA analyses. (E) Heatmap of RNA polymerase II subunit expression levels across indicated conditions. (F) Western blot of Pol II, NSP1, Flag-NSP1, GAPDH, and β-actin protein levels. (G and H) Immunoblotting of Pol II and Flag after immunoprecipitation with anti-Flag (G) and anti-Pol II (H). IgG served as IP control; Input = 5% of total lysate.
