# Supplementary material for: SARS-CoV-2 nonstructural protein 1 suppresses host transcription by reducing RNA polymerase II levels
Source: iScience. 2025 Nov 26;28(12):114233. doi: 10.1016/j.isci.2025.114233 (PMC12741407; doi:10.1016/j.isci.2025.114233)
Supplement: Data S1. The sequences of constructed plasmids [file mmc2.pdf]

5′ GACATTGATTATTGACTAGTTATTAATAGTAATCAATTACGGGGTCATTAGTTCATAGCCCATATATGGAGTTCCGCGTT 80
3′ CTGTAACTAATAACTGATCAATAATTATCATTAGTTAATGCCCCAGTAATCAAGTATCGGGTATATACCTCAAGGCGCAA
CMV enhancer

ACATAACTTACGGTAAATGGCCCGCCTGGCTGACCGCCCAACGACCCCCGCCCATTGACGTCAATAATGACGTATGTTCC 160
TGTATTGAATGCCATTTACCGGGCGGACCGACTGGCGGGTTGCTGGGGGCGGGTAACTGCAGTTATTACTGCATACAAGG
CMV enhancer

CATAGTAACGCCAATAGGGACTTTCCATTGACGTCAATGGGTGGAGTATTTACGGTAAACTGCCCACTTGGCAGTACATC 240
GTATCATTGCGGTTATCCCTGAAAGGTAACTGCAGTTACCCACCTCATAAATGCCATTTGACGGGTGAACCGTCATGTAG
CMV enhancer

AAGTGTATCATATGCCAAGTACGCCCCCTATTGACGTCAATGACGGTAAATGGCCCGCCTGGCATTATGCCCAGTACATG 320
TTCACATAGTATACGGTTCATGCGGGGGATAACTGCAGTTACTGCCATTTACCGGGCGGACCGTAATACGGGTCATGTAC
CMV enhancer

ACCTTATGGGACTTTCCTACTTGGCAGTACATCTACGTATTAGTCATCGCTATTACCATGGTGATGCGGTTTTGGCAGTA 400
TGGAATACCCTGAAAGGATGAACCGTCATGTAGATGCATAATCAGTAGCGATAATGGTACCACTACGCCAAAACCGTCAT
CMV enhancer | CMV promoter

CATCAATGGGCGTGGATAGCGGTTTGACTCACGGGGATTTCCAAGTCTCCACCCCATTGACGTCAATGGGAGTTTGTTTT 480
GTAGTTACCCGCACCTATCGCCAAACTGAGTGCCCCTAAAGGTTCAGAGGTGGGGTAACTGCAGTTACCCTCAAACAAAA
CMV promoter

GGAACCAAAATCAACGGGACTTTCCAAAATGTCGTAACAACTCCGCCCCATTGACGCAAATGGGCGGTAGGCGTGTACGG 560
CCTTGGTTTTAGTTGCCCTGAAAGGTTTTACAGCATTGTTGAGGCGGGGTAACTGCGTTTACCCGCCATCCGCACATGCC
CMV promoter

TGGGAGGTCTATATAAGCAGAGCTATTAAAGGTTTATACCTTCCCAGGTAACAAACCAACCAACTTTCGATCTCTTGTAG 640
ACCCTCCAGATATATTCGTCTCGATAATTTCCAAATATGGAAGGGTCCATTGTTTGGTTGGTTGAAAGCTAGAGAACATC
CMV promoter | leader

ATCTGTTCTCTAAACGAACATGGACTACAAAGACGATGACGACAAGGGATCCACTAGTAACGGCCGCCAGTGTGCTGGAA 720
TAGACAAGAGATTTGCTTGTACCTGATGTTTCTGCTACTGCTGTTCCCTAGGTGATCATTGCCGGCGGTCACACGACCTT
leader | FLAG

TTCTGCAGATATCCAGCACAGTGGCGGCCGCTCGAGTCTAGAGGGCCCGCGGTTCGAACAAAAACTCATCTCAGAAGAGG 800
AAGACGTCTATAGGTCGTGTCACCGCCGGCGAGCTCAGATCTCCCGGGCGCCAAGCTTGTTTTTGAGTAGAGTCTTCTCC

ATCTGAATATGCATACCGGTCATCATCACCATCACCATTGAGTCCCCTCTCCCTCCCCCCCCCCTAACGTTACTGGCCGA 880
TAGACTTATACGTATGGCCAGTAGTAGTGGTAGTGGTAACTCAGGGGAGAGGGAGGGGGGGGGGATTGCAATGACCGGCT
IRES2

AGCCGCTTGGAATAAGGCCGGTGTGCGTTTGTCTATATGTTATTTTCCACCATATTGCCGTCTTTTGGCAATGTGAGGGC 960
TCGGCGAACCTTATTCCGGCCACACGCAAACAGATATACAATAAAAGGTGGTATAACGGCAGAAAACCGTTACACTCCCG
IRES2

CCGGAAACCTGGCCCTGTCTTCTTGACGAGCATTCCTAGGGGTCTTTCCCCTCTCGCCAAAGGAATGCAAGGTCTGTTGA 1040
GGCCTTTGGACCGGGACAGAAGAACTGCTCGTAAGGATCCCCAGAAAGGGGAGAGCGGTTTCCTTACGTTCCAGACAACT

IRES2

ATGTCGTGAAGGAAGCAGTTCCTCTGGAAGCTTCTTGAAGACAAACAACGTCTGTAGCGACCCTTTGCAGGCAGCGGAAC 1120
TACAGCACTTCCTTCGTCAAGGAGACCTTCGAAGAACTTCTGTTTGTTGCAGACATCGCTGGGAAACGTCCGTCGCCTTG

IRES2

CCCCCACCTGGCGACAGGTGCCTCTGCGGCCAAAAGCCACGTGTATAAGATACACCTGCAAAGGCGGCACAACCCCAGTGC 1200
GGGGGTGGACCGCTGTCCACGGAGACGCCGGTTTTCGGTGCACATATTCTATGTGGACGTTTCCGCCGTGTTGGGGTCACG

IRES2

CACGTTGTGAGTTGGATAGTTGTGGAAAGAGTCAAATGGCTCTCCTCAAgCGtAttcAACAAGGGGCTGAAGGATGCCCA 1280
GTGCAACACTCAACCTATCAACACCTTTCTCAGTTTACCGAGAGGAGTTcGCaTaagTTGTTCCCCGACTTCCTACGGGT

IRES2

GAAGGTACCCCATTGTATGGGATCTGATCTGGGGCCTCGGTACACATGCTTTACATGTGTTTAGTCGAGGTTAAAAAAAC 1360
CTTCCATGGGGTAACATACCCTAGACTAGACCCCGGAGCCATGTGTACGAAATGTACACAAATCAGCTCCAATTTTTTTG

IRES2

GTCTAGGCCCCCCGAACCACGGGGACGTGGTTTTCCTTTGAAAAACACGATGATAATATGGCCACAACCATGGTGAGCAA 1440
CAGATCCGGGGGGCTTGGTGCCCCTGCACCAAAAGGAAACTTTTTGTGCTACTATTATACCGGTGTTGGTACCACTCGTT

ATG 1a

IRES2 EGFP

GGGCGAGGAGCTGTTCACCGGGGTGGTGCCCATCCTGGTCGAGCTGGACGGCGACGTAAACGACCACAAGTTCAGCGTGT 1520
CCCGCTCCTCGACAAGTGGCCCCACCACGGGTAGGACCAGCTCGACCTGCCGCTGCATTTGCTGGTGTTCAAGTCGCACA

EGFP

CCGGCGAGGGCGAGGGCGATGCCACCTACGGCAAGCTGACCCTGAAGTTCATCTGCACCACCGGCAaGCTGCCCGTGCCC 1600
GGCCGCTCCCGCTCCCGCTACGGTGGATGCCGTTCGACTGGGACTTCAAGTAGACGTGGTGGCCGTtCGACGGGCACGGG

EGFP

TGGCCcAcCCTCGTGACCACCCTGAcCTACGGCGTGCAGTGCTTCAGCCGCTAcCCCGACCACATGAAGCAGCACGACTT 1680
ACCGGgTgGGAGCACTGGTGGGACTgGATGCCGCACGTCACGAAGTCGGCGATgGGGCTGGTGTACTTCGTCGTGCTGAA

EGFP

CTTCAAGTCCGCCATGCCCGAAGGCTACGTCCAGGAGCGCACCATCTTCTTCAAGGACGACGGCAACTACAAGACCCGCG 1760
GAAGTTCAGGCGGTACGGGCTTCCGATGCAGGTCCTCGCGTGGTAGAAGAAGTTCCTGCTGCCGTTGATGTTCTGGGCGC

EGFP

CCGAGGTGaAGTTCGAGGGCGACACCCTGGTGAACCGCATCGAGCTGAAGGGCATCGACTTCAAGGAGGACGGCAACATC 1840
GGCTCCACtTCAAGCTCCCGCTGTGGGACCACTTGGCGTAGCTCGACTTCCCGTAGCTGAAGTTCCTCCTGCCGTTGTAG

EGFP

CTGGGGCACAAGCTGGAGTACAACTACAACAGCCACAACGTCTATATCATGGCCGACAAGCAGAAGAACGGCATCAAGGT 1920
GACCCCGTGTTCGACCTCATGTTGATGTTGTCGGTGTTGCAGATATAGTACCGGCTGTTCGTCTTCTTGCCGTAGTTCCA

EGFP

GAACTTCAAGATCCGCCACAACATCGAGGACGGCAGCGTGCAGCTCGCCGACCACTACCAGCAGAACACCCCCATCGGCG 2000
CTTGAAGTTCTAGGCGGTGTTGTAGCTCCTGCCGTCGCACGTCGAGCGGCTGGTGATGGTCGTCTTGTGGGGGTAGCCGC

EGFP

ACGGCCCCGTGCTGCTGCCCGACAACCACTACCTGAGCACCCAGTCCGCCCTGAGCAAAGACCCCAACGAGAAGCGCGAT 2080
TGCCGGGGCACGACGACGGGCTGTTGGTGATGGACTCGTGGGTCAGGCGGGACTCGTTTCTGGGGTTGCTCTTCGCGCTA

EGFP

CACATGGTCCTGCTGGAGTTCGTGACCGCCGCCGGGATCACTCTCGGCATGGACGAGCTGTACAAGTAA 3′ 2149
GTGTACCAGGACGACCTCAAGCACTGGCGGCGGCCCTAGTGAGAGCCGTACCTGCTCGACATGTTCATT 5′

EGFP

	Feature	Location	Size	Color	Directionality	Type
✓	**CMV enhancer**	1 .. 380	380 bp		⊢⊣	enhancer
	/note = human cytomegalovirus immediate early enhancer					
✓	**CMV promoter**	381 .. 584	204 bp		→	promoter
	/note = human cytomegalovirus (CMV) immediate early promoter					
✓	**leader**	585 .. 659	75 bp		⊢⊣	misc_feature
✓	**FLAG**	663 .. 686	24 bp		→	misc_feature
✓	**IRES2**	844 .. 1429	586 bp		⊢⊣	misc_feature
	▸ 3 segments					
	/note = internal ribosome entry site (IRES) of the encephalomyocarditis virus (EMCV)					
✓	**EGFP**	1430 .. 2149	720 bp		→	CDS
	▸ 3 segments					
	/note = mammalian codon-optimized					

5′ GACATTGATTATTGACTAGTTATTAATAGTAATCAATTACGGGGTCATTAGTTCATAGCCCATATATGGAGTTCCGCGTT 80
3′ CTGTAACTAATAACTGATCAATAATTATCATTAGTTAATGCCCCAGTAATCAAGTATCGGGTATATACCTCAAGGCGCAA
CMV enhancer

ACATAACTTACGGTAAATGGCCCGCCTGGCTGACCGCCCAACGACCCCCGCCCATTGACGTCAATAATGACGTATGTTCC 160
TGTATTGAATGCCATTTACCGGGCGGACCGACTGGCGGGTTGCTGGGGGCGGGTAACTGCAGTTATTACTGCATACAAGG
CMV enhancer

CATAGTAACGCCAATAGGGACTTTCCATTGACGTCAATGGGTGGAGTATTTACGGTAAACTGCCCACTTGGCAGTACATC 240
GTATCATTGCGGTTATCCCTGAAAGGTAACTGCAGTTACCCACCTCATAAATGCCATTTGACGGGTGAACCGTCATGTAG
CMV enhancer

AAGTGTATCATATGCCAAGTACGCCCCCTATTGACGTCAATGACGGTAAATGGCCCGCCTGGCATTATGCCCAGTACATG 320
TTCACATAGTATACGGTTCATGCGGGGGATAACTGCAGTTACTGCCATTTACCGGGCGGACCGTAATACGGGTCATGTAC
CMV enhancer

ACCTTATGGGACTTTCCTACTTGGCAGTACATCTACGTATTAGTCATCGCTATTACCATGGTGATGCGGTTTTGGCAGTA 400
TGGAATACCCTGAAAGGATGAACCGTCATGTAGATGCATAATCAGTAGCGATAATGGTACCACTACGCCAAAACCGTCAT
CMV enhancer | CMV promoter

CATCAATGGGCGTGGATAGCGGTTTGACTCACGGGGATTTCCAAGTCTCCACCCCATTGACGTCAATGGGAGTTTGTTTT 480
GTAGTTACCCGCACCTATCGCCAAACTGAGTGCCCCTAAAGGTTCAGAGGTGGGGTAACTGCAGTTACCCTCAAACAAAA
CMV promoter

GGAACCAAAATCAACGGGACTTTCCAAAATGTCGTAACAACTCCGCCCCATTGACGCAAATGGGCGGTAGGCGTGTACGG 560
CCTTGGTTTTAGTTGCCCTGAAAGGTTTTACAGCATTGTTGAGGCGGGGTAACTGCGTTTACCCGCCATCCGCACATGCC
CMV promoter

TGGGAGGTCTATATAAGCAGAGCTATTAAAGGTTTATACCTTCCCAGGTAACAAACCAACCAACTTTCGATCTCTTGTAG 640
ACCCTCCAGATATATTCGTCTCGATAATTTCCAAATATGGAAGGGTCCATTGTTTGGTTGGTTGAAAGCTAGAGAACATC
CMV promoter | leader

ATCTGTTCTCTAAACGAACATGGACTACAAAGACGATGACGACAAGGGATCCACTAGTAACGGCCGCCAGTGTGCTGAAT 720
TAGACAAGAGATTTGCTTGTACCTGATGTTTCTGCTACTGCTGTTCCCTAGGTGATCATTGCCGGCGGTCACACGACTTA
leader | FLAG

TCATGGAGAGCCTTGTCCCTGGTTTCAACGAGAAAACACACGTCCAACTCAGTTTGCCTGTTTTACAGGTTCGCGACGTG 800
AGTACCTCTCGGAACAGGGACCAAAGTTGCTCTTTTGTGTGCAGGTTGAGTCAAACGGACAAAATGTCCAAGCGCTGCAC
NSP1

CTCGTACGTGGCTTTGGAGACTCCGTGGAGGAGGTCTTATCAGAGGCACGTCAACATCTTAAAGATGGCACTTGTGGCTT 880
GAGCATGCACCGAAACCTCTGAGGCACCTCCTCCAGAATAGTCTCCGTGCAGTTGTAGAATTTCTACCGTGAACACCGAA
NSP1

AGTAGAAGTTGAAAAAGGCGTTTTGCCTCAACTTGAACAGCCCTATGTGTTCATCAAACGTTCGGATGCTCGAACTGCAC 960
TCATCTTCAACTTTTTCCGCAAAACGGAGTTGAACTTGTCGGGATACACAAGTAGTTTGCAAGCCTACGAGCTTGACGTG
NSP1

CTCATGGTCATGTTATGGTTGAGCTGGTAGCAGAACTCGAAGGCATTCAGTACGGTCGTAGTGGTGAGACACTTGGTGTC 1040
GAGTACCAGTACAATACCAACTCGACCATCGTCTTGAGCTTCCGTAAGTCATGCCAGCATCACCACTCTGTGAACCACAG
NSP1

CTTGTCCCTCATGTGGGCGAAATACCAGTGGCTTACCGCAAGGTTCTTCTTCGTAAGAACGGTAATAAAGGAGCTGGTGG 1120
GAACAGGGAGTACACCCGCTTTATGGTCACCGAATGGCGTTCCAAGAAGAAGCATTCTTGCCATTATTTCCTCGACCACC
NSP1

CCATAGTTACGGCGCCGATCTAAAGTCATTTGACTTAGGCGACGAGCTTGGCACTGATCCTTATGAAGATTTTCAAGAAA 1200
GGTATCAATGCCGCGGCTAGATTTCAGTAAACTGAATCCGCTGCTCGAACCGTGACTAGGAATACTTCTAAAAGTTCTTT
NSP1

ACTGGAACACTAAACATAGCAGTGGTGTTACCCGTGAACTCATGCGTGAGCTTAACGGAGGGTAATCTAGAGGGCCCGCG 1280
TGACCTTGTGATTTGTATCGTCACCACAATGGGCACTTGAGTACGCACTCGAATTGCCTCCCATTAGATCTCCCGGGCGC
NSP1

GTTCGAACAAAAACTCATCTCAGAAGAGGATCTGAATATGCATACCGGTCATCATCACCATCACCATTGAGTCCCCTCTC 1360
CAAGCTTGTTTTTGAGTAGAGTCTTCTCCTAGACTTATACGTATGGCCAGTAGTAGTGGTAGTGGTAACTCAGGGGAGAG
IRES2

CCTCCCCCCCCCCTAACGTTACTGGCCGAAGCCGCTTGGAATAAGGCCGGTGTGCGTTTGTCTATATGTTATTTTCCACC 1440
GGAGGGGGGGGGGATTGCAATGACCGGCTTCGGCGAACCTTATTCCGGCCACACGCAAACAGATATACAATAAAAGGTGG
IRES2

ATATTGCCGTCTTTTGGCAATGTGAGGGCCCGGAAACCTGGCCCTGTCTTCTTGACGAGCATTCCTAGGGGTCTTTCCCC 1520
TATAACGGCAGAAAACCGTTACACTCCCGGGCCTTTGGACCGGGACAGAAGAACTGCTCGTAAGGATCCCCAGAAAGGGG
IRES2

TCTCGCCAAAGGAATGCAAGGTCTGTTGAATGTCGTGAAGGAAGCAGTTCCTCTGGAAGCTTCTTGAAGACAAACAACGT 1600
AGAGCGGTTTCCTTACGTTCCAGACAACTTACAGCACTTCCTTCGTCAAGGAGACCTTCGAAGAACTTCTGTTTGTTGCA
IRES2

CTGTAGCGACCCTTTGCAGGCAGCGGAACCCCCCACCTGGCGACAGGTGCCTCTGCGGCCAAAAGCCACGTGTATAAGATA 1680
GACATCGCTGGGAAACGTCCGTCGCCTTGGGGGGTGGACCGCTGTCCACGGAGACGCCGGTTTTCGGTGCACATATTCTAT
IRES2

CACCTGCAAAGGCGGCACAACCCCAGTGCCACGTTGTGAGTTGGATAGTTGTGGAAAGAGTCAAATGGCTCTCCTCAAgC 1760
GTGGACGTTTCCGCCGTGTTGGGGTCACGGTGCAACACTCAACCTATCAACACCTTTCTCAGTTTACCGAGAGGAGTTcG
IRES2

GtAttcAACAAGGGGCTGAAGGATGCCCAGAAGGTACCCCATTGTATGGGATCTGATCTGGGGCCTCGGTACACATGCTT 1840
CaTaagTTGTTCCCCGACTTCCTACGGGTCTTCCATGGGGTAACATACCCTAGACTAGACCCCGGAGCCATGTGTACGAA
IRES2

TACATGTGTTTAGTCGAGGTTAAAAAAACGTCTAGGCCCCCCGAACCACGGGGACGTGGTTTTCCTTTGAAAAACACGAT 1920
ATGTACACAAATCAGCTCCAATTTTTTTGCAGATCCGGGGGGCTTGGTGCCCCTGCACCAAAAGGAAACTTTTTGTGCTA

IRES2

GATAATATGGCCACAACCATGGTGAGCAAGGGCGAGGAGCTGTTCACCGGGGTGGTGCCCATCCTGGTCGAGCTGGACGG 2000
CTATTATACCGGTGTTGGTACCACTCGTTCCCGCTCCTCGACAAGTGGCCCCACCACGGGTAGGACCAGCTCGACCTGCC

ATG 1a
IRES2 EGFP

CGACGTAAACGACCACAAGTTCAGCGTGTCCGGCGAGGGCGAGGGCGATGCCACCTACGGCAAGCTGACCCTGAAGTTCA 2080
GCTGCATTTGCTGGTGTTCAAGTCGCACAGGCCGCTCCCGCTCCCGCTACGGTGGATGCCGTTCGACTGGGACTTCAAGT

EGFP

TCTGCACCACCGGCAaGCTGCCCGTGCCCTGGCCcAcCCTCGTGACCACCCTGAcCTACGGCGTGCAGTGCTTCAGCCGC 2160
AGACGTGGTGGCCGTtCGACGGGCACGGGACCGGgTgGGAGCACTGGTGGGACTgGATGCCGCACGTCACGAAGTCGGCG

EGFP

TAcCCCGACCACATGAAGCAGCACGACTTCTTCAAGTCCGCCATGCCCGAAGGCTACGTCCAGGAGCGCACCATCTTCTT 2240
ATgGGGCTGGTGTACTTCGTCGTGCTGAAGAAGTTCAGGCGGTACGGGCTTCCGATGCAGGTCCTCGCGTGGTAGAAGAA

EGFP

CAAGGACGACGGCAACTACAAGACCCGCGCCGAGGTGaAGTTCGAGGGCGACACCCTGGTGAACCGCATCGAGCTGAAGG 2320
GTTCCTGCTGCCGTTGATGTTCTGGGCGCGGCTCCACtTCAAGCTCCCGCTGTGGGACCACTTGGCGTAGCTCGACTTCC

EGFP

GCATCGACTTCAAGGAGGACGGCAACATCCTGGGGCACAAGCTGGAGTACAACTACAACAGCCACAACGTCTATATCATG 2400
CGTAGCTGAAGTTCCTCCTGCCGTTGTAGGACCCCGTGTTCGACCTCATGTTGATGTTGTCGGTGTTGCAGATATAGTAC

EGFP

GCCGACAAGCAGAAGAACGGCATCAAGGTGAACTTCAAGATCCGCCACAACATCGAGGACGGCAGCGTGCAGCTCGCCGA 2480
CGGCTGTTCGTCTTCTTGCCGTAGTTCCACTTGAAGTTCTAGGCGGTGTTGTAGCTCCTGCCGTCGCACGTCGAGCGGCT

EGFP

CCACTACCAGCAGAACACCCCCATCGGCGACGGCCCCGTGCTGCTGCCCGACAACCACTACCTGAGCACCCAGTCCGCCC 2560
GGTGATGGTCGTCTTGTGGGGGTAGCCGCTGCCGGGGCACGACGACGGGCTGTTGGTGATGGACTCGTGGGTCAGGCGGG

EGFP

TGAGCAAAGACCCCAACGAGAAGCGCGATCACATGGTCCTGCTGGAGTTCGTGACCGCCGCCGGGATCACTCTCGGCATG 2640
ACTCGTTTCTGGGGTTGCTCTTCGCGCTAGTGTACCAGGACGACCTCAAGCACTGGCGGCGGCCCTAGTGAGAGCCGTAC

EGFP

GACGAGCTGTACAAGTAA 3′
2658
CTGCTCGACATGTTCATT 5′

EGFP

Feature		Location	Size			Type
✓ **CMV enhancer**		1 .. 380	380 bp			enhancer
/note	= human cytomegalovirus immediate early enhancer					
✓ **CMV promoter**		381 .. 584	204 bp		→	promoter
/note	= human cytomegalovirus (CMV) immediate early promoter					
✓ **leader**		585 .. 659	75 bp			misc_feature
✓ **FLAG**		663 .. 686	24 bp		→	misc_feature
/product	= FLAG® epitope tag, followed by an enterokinase cleavage site					
✓ **NSP1**		723 .. 1265	543 bp			misc_feature
✓ **IRES2**		1353 .. 1938	586 bp			misc_feature
3 segments						
/note	= internal ribosome entry site (IRES) of the encephalomyocarditis virus (EMCV)					
✓ **EGFP**		1939 .. 2658	720 bp		→	CDS
3 segments						
/note	= mammalian codon-optimized					

```
5′ GACATTGATTATTGACTAGTTATTAATAGTAATCAATTACGGGGTCATTAGTTCATAGCCCATATATGGAGTTCCGCGTT   80
3′ CTGTAACTAATAACTGATCAATAATTATCATTAGTTAATGCCCCAGTAATCAAGTATCGGGTATATACCTCAAGGCGCAA
   CMV enhancer

   ACATAACTTACGGTAAATGGCCCGCCTGGCTGACCGCCCAACGACCCCCGCCCATTGACGTCAATAATGACGTATGTTCC   160
   TGTATTGAATGCCATTTACCGGGCGGACCGACTGGCGGGTTGCTGGGGGCGGGTAACTGCAGTTATTACTGCATACAAGG
   CMV enhancer

   CATAGTAACGCCAATAGGGACTTTCCATTGACGTCAATGGGTGGAGTATTTACGGTAAACTGCCCACTTGGCAGTACATC   240
   GTATCATTGCGGTTATCCCTGAAAGGTAACTGCAGTTACCCACCTCATAAATGCCATTTGACGGGTGAACCGTCATGTAG
   CMV enhancer

   AAGTGTATCATATGCCAAGTACGCCCCCTATTGACGTCAATGACGGTAAATGGCCCGCCTGGCATTATGCCCAGTACATG   320
   TTCACATAGTATACGGTTCATGCGGGGGATAACTGCAGTTACTGCCATTTACCGGGCGGACCGTAATACGGGTCATGTAC
   CMV enhancer

   ACCTTATGGGACTTTCCTACTTGGCAGTACATCTACGTATTAGTCATCGCTATTACCATGGTGATGCGGTTTTGGCAGTA   400
   TGGAATACCCTGAAAGGATGAACCGTCATGTAGATGCATAATCAGTAGCGATAATGGTACCACTACGCCAAAACCGTCAT
   CMV enhancer                                    CMV promoter

   CATCAATGGGCGTGGATAGCGGTTTGACTCACGGGGATTTCCAAGTCTCCACCCCATTGACGTCAATGGGAGTTTGTTTT   480
   GTAGTTACCCGCACCTATCGCCAAACTGAGTGCCCCTAAAGGTTCAGAGGTGGGGTAACTGCAGTTACCCTCAAACAAAA
   CMV promoter

   GGAACCAAAATCAACGGGACTTTCCAAAATGTCGTAACAACTCCGCCCCATTGACGCAAATGGGCGGTAGGCGTGTACGG   560
   CCTTGGTTTTAGTTGCCCTGAAAGGTTTTACAGCATTGTTGAGGCGGGGTAACTGCGTTTACCCGCCATCCGCACATGCC
   CMV promoter

   TGGGAGGTCTATATAAGCAGAGCTATTAAAGGTTTATACCTTCCCAGGTAACAAACCAACCAACTTTCGATCTCTTGTAG   640
   ACCCTCCAGATATATTCGTCTCGATAATTTCCAAATATGGAAGGGTCCATTGTTTGGTTGGTTGAAAGCTAGAGAACATC
   CMV promoter   leader

   ATCTGTTCTCTAAACGAACATGGACTACAAAGACGATGACGACAAGGGATCCACTAGTAACGGCCGCCAGTGTGCTGAAT   720
   TAGACAAGAGATTTGCTTGTACCTGATGTTTCTGCTACTGCTGTTCCCTAGGTGATCATTGCCGGCGGTCACACGACTTA
   leader   FLAG

   TCATGGAGAGCCTTGTCCCTGGTTTCAACGAGAAAACACACGTCCAACTCAGTTTGCCTGTTTTACAGGTTCGCGACGTG   800
   AGTACCTCTCGGAACAGGGACCAAAGTTGCTCTTTTGTGTGCAGGTTGAGTCAAACGGACAAAATGTCCAAGCGCTGCAC
   NSP1-KH

   CTCGTACGTGGCTTTGGAGACTCCGTGGAGGAGGTCTTATCAGAGGCACGTCAACATCTTAAAGATGGCACTTGTGGCTT   880
   GAGCATGCACCGAAACCTCTGAGGCACCTCCTCCAGAATAGTCTCCGTGCAGTTGTAGAATTTCTACCGTGAACACCGAA
   NSP1-KH

   AGTAGAAGTTGAAAAAGGCGTTTTGCCTCAACTTGAACAGCCCTATGTGTTCATCAAACGTTCGGATGCTCGAACTGCAC   960
   TCATCTTCAACTTTTTCCGCAAAACGGAGTTGAACTTGTCGGGATACACAAGTAGTTTGCAAGCCTACGAGCTTGACGTG
   NSP1-KH
```

CTCATGGTCATGTTATGGTTGAGCTGGTAGCAGAACTCGAAGGCATTCAGTACGGTCGTAGTGGTGAGACACTTGGTGTC 1040
GAGTACCAGTACAATACCAACTCGACCATCGTCTTGAGCTTCCGTAAGTCATGCCAGCATCACCACTCTGTGAACCACAG
NSP1-KH

CTTGTCCCTCATGTGGGCGAAATACCAGTGGCTTACCGCAAGGTTCTTCTTCGTAAGAACGGTAATAAAGGAGCTGGTGG 1120
GAACAGGGAGTACACCCGCTTTATGGTCACCGAATGGCGTTCCAAGAAGAAGCATTCTTGCCATTATTTCCTCGACCACC
NSP1-KH

CCATAGTTACGGCGCCGATCTAAAGTCATTTGACTTAGGCGACGAGCTTGGCACTGATCCTTATGAAGATTTTCAAGAAA 1200
GGTATCAATGCCGCGGCTAGATTTCAGTAAACTGAATCCGCTGCTCGAACCGTGACTAGGAATACTTCTAAAAGTTCTTT
NSP1-KH

ACTGGAACACTGCAGCTAGCAGTGGTGTTACCCGTGAACTCATGCGTGAGCTTAACGGAGGGTAATCTAGAGGGCCCGCG 1280
TGACCTTGTGACGTCGATCGTCACCACAATGGGCACTTGAGTACGCACTCGAATTGCCTCCCATTAGATCTCCCGGGCGC
NSP1-KH
K164A/H165A

GTTCGAACAAAAACTCATCTCAGAAGAGGATCTGAATATGCATACCGGTCATCATCACCATCACCATTGAGTCCCCTCTC 1360
CAAGCTTGTTTTTGAGTAGAGTCTTCTCCTAGACTTATACGTATGGCCAGTAGTAGTGGTAGTGGTAACTCAGGGGAGAG
IRES2

CCTCCCCCCCCCCTAACGTTACTGGCCGAAGCCGCTTGGAATAAGGCCGGTGTGCGTTTGTCTATATGTTATTTTCCACC 1440
GGAGGGGGGGGGGATTGCAATGACCGGCTTCGGCGAACCTTATTCCGGCCACACGCAAACAGATATACAATAAAAGGTGG
IRES2

ATATTGCCGTCTTTTGGCAATGTGAGGGCCCGGAAACCTGGCCCTGTCTTCTTGACGAGCATTCCTAGGGGTCTTTCCCC 1520
TATAACGGCAGAAAACCGTTACACTCCCGGGCCTTTGGACCGGGACAGAAGAACTGCTCGTAAGGATCCCCAGAAAGGGG
IRES2

TCTCGCCAAAGGAATGCAAGGTCTGTTGAATGTCGTGAAGGAAGCAGTTCCTCTGGAAGCTTCTTGAAGACAAACAACGT 1600
AGAGCGGTTTCCTTACGTTCCAGACAACTTACAGCACTTCCTTCGTCAAGGAGACCTTCGAAGAACTTCTGTTTGTTGCA
IRES2

CTGTAGCGACCCTTTGCAGGCAGCGGAACCCCCCACCTGGCGACAGGTGCCTCTGCGGCCAAAGCCACGTGTATAAGATA 1680
GACATCGCTGGGAAACGTCCGTCGCCTTGGGGGGTGGACCGCTGTCCACGGAGACGCCGGTTTCGGTGCACATATTCTAT
IRES2

CACCTGCAAAGGCGGCACAACCCCAGTGCCACGTTGTGAGTTGGATAGTTGTGGAAAGAGTCAAATGGCTCTCCTCAAgC 1760
GTGGACGTTTCCGCCGTGTTGGGGTCACGGTGCAACACTCAACCTATCAACACCTTTCTCAGTTTACCGAGAGGAGTTcG
IRES2

GtAttcAACAAGGGGCTGAAGGATGCCCAGAAGGTACCCCATTGTATGGGATCTGATCTGGGGCCTCGGTACACATGCTT 1840
CaTaagTTGTTCCCCGACTTCCTACGGGTCTTCCATGGGGTAACATACCCTAGACTAGACCCCGGAGCCATGTGTACGAA
IRES2

```
TACATGTGTTTAGTCGAGGTTAAAAAAACGTCTAGGCCCCCCGAACCACGGGGACGTGGTTTTCCTTTGAAAAACACGAT   1920
ATGTACACAAATCAGCTCCAATTTTTTTGCAGATCCGGGGGGCTTGGTGCCCCTGCACCAAAAGGAAACTTTTTGTGCTA
IRES2

GATAATATGGCCACAACCATGGTGAGCAAGGGCGAGGAGCTGTTCACCGGGGTGGTGCCCATCCTGGTCGAGCTGGACGG   2000
CTATTATACCGGTGTTGGTACCACTCGTTCCCGCTCCTCGACAAGTGGCCCCACCACGGGTAGGACCAGCTCGACCTGCC
ATG  1a
IRES2  EGFP

CGACGTAAACGACCACAAGTTCAGCGTGTCCGGCGAGGGCGAGGGCGATGCCACCTACGGCAAGCTGACCCTGAAGTTCA   2080
GCTGCATTTGCTGGTGTTCAAGTCGCACAGGCCGCTCCCGCTCCCGCTACGGTGGATGCCGTTCGACTGGGACTTCAAGT
EGFP

TCTGCACCACCGGCAaGCTGCCCGTGCCCTGGCCcAcCCTCGTGACCACCCTGAcCTACGGCGTGCAGTGCTTCAGCCGC   2160
AGACGTGGTGGCCGTtCGACGGGCACGGGACCGGgTgGGAGCACTGGTGGGACTgGATGCCGCACGTCACGAAGTCGGCG
EGFP

TAcCCCGACCACATGAAGCAGCACGACTTCTTCAAGTCCGCCATGCCCGAAGGCTACGTCCAGGAGCGCACCATCTTCTT   2240
ATgGGGCTGGTGTACTTCGTCGTGCTGAAGAAGTTCAGGCGGTACGGGCTTCCGATGCAGGTCCTCGCGTGGTAGAAGAA
EGFP

CAAGGACGACGGCAACTACAAGACCCGCGCCGAGGTGaAGTTCGAGGGCGACACCCTGGTGAACCGCATCGAGCTGAAGG   2320
GTTCCTGCTGCCGTTGATGTTCTGGGCGCGGCTCCACtTCAAGCTCCCGCTGTGGGACCACTTGGCGTAGCTCGACTTCC
EGFP

GCATCGACTTCAAGGAGGACGGCAACATCCTGGGGCACAAGCTGGAGTACAACTACAACAGCCACAACGTCTATATCATG   2400
CGTAGCTGAAGTTCCTCCTGCCGTTGTAGGACCCCGTGTTCGACCTCATGTTGATGTTGTCGGTGTTGCAGATATAGTAC
EGFP

GCCGACAAGCAGAAGAACGGCATCAAGGTGAACTTCAAGATCCGCCACAACATCGAGGACGGCAGCGTGCAGCTCGCCGA   2480
CGGCTGTTCGTCTTCTTGCCGTAGTTCCACTTGAAGTTCTAGGCGGTGTTGTAGCTCCTGCCGTCGCACGTCGAGCGGCT
EGFP

CCACTACCAGCAGAACACCCCCATCGGCGACGGCCCCGTGCTGCTGCCCGACAACCACTACCTGAGCACCCAGTCCGCCC   2560
GGTGATGGTCGTCTTGTGGGGGTAGCCGCTGCCGGGGCACGACGACGGGCTGTTGGTGATGGACTCGTGGGTCAGGCGGG
EGFP

TGAGCAAAGACCCCAACGAGAAGCGCGATCACATGGTCCTGCTGGAGTTCGTGACCGCCGCCGGGATCACTCTCGGCATG   2640
ACTCGTTTCTGGGGTTGCTCTTCGCGCTAGTGTACCAGGACGACCTCAAGCACTGGCGGCGGCCCTAGTGAGAGCCGTAC
EGFP

GACGAGCTGTACAAGTAA   3′
                    2658
CTGCTCGACATGTTCATT   5′
EGFP
```

	Feature	Location	Size			Type
✓	**CMV enhancer**	1 .. 380	380 bp			enhancer
	/note	= human cytomegalovirus immediate early enhancer				
✓	**CMV promoter**	381 .. 584	204 bp		→	promoter
	/note	= human cytomegalovirus (CMV) immediate early promoter				
✓	**leader**	585 .. 659	75 bp			misc_feature
✓	**FLAG**	663 .. 686	24 bp		→	misc_feature
	/product	= FLAG® epitope tag, followed by an enterokinase cleavage site				
✓	**NSP1-KH**	723 .. 1265	543 bp			misc_feature
✓	**K164A/H165A**	1212 .. 1217	6 bp			misc_feature
✓	**IRES2**	1353 .. 1938	586 bp			misc_feature
	3 segments					
	/note	= internal ribosome entry site (IRES) of the encephalomyocarditis virus (EMCV)				
✓	**EGFP**	1939 .. 2658	720 bp		→	CDS
	3 segments					
	/note	= mammalian codon-optimized				
