# Supplementary material for: SARS-CoV-2 nonstructural protein 1 suppresses host transcription by reducing RNA polymerase II levels
Source: iScience. 2025 Nov 26;28(12):114233. doi: 10.1016/j.isci.2025.114233 (PMC12741407; doi:10.1016/j.isci.2025.114233)

Raw data of WB.The red rectangle highlights the lanes presented in the manuscript.

Figure 1D

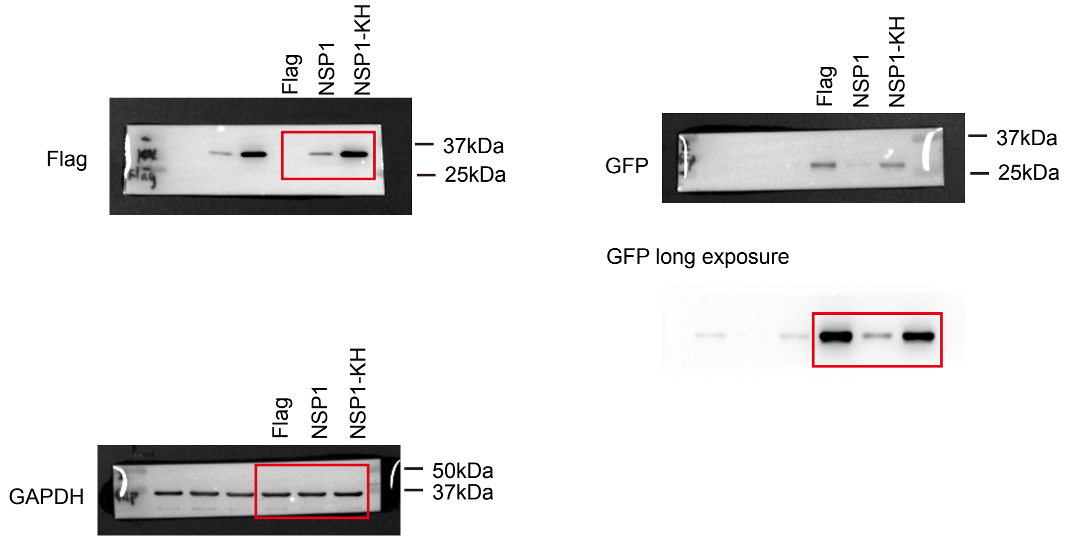

Figure 5C

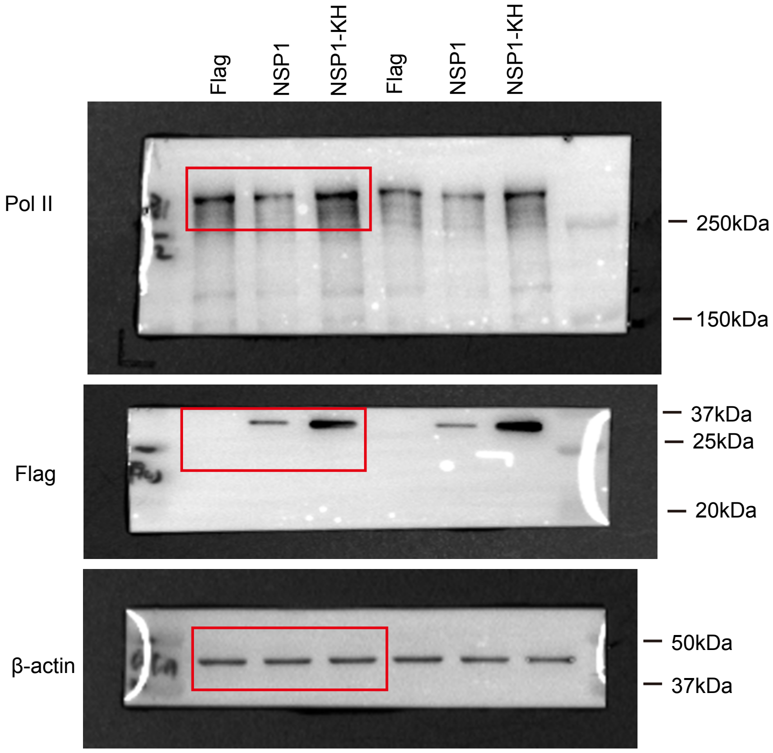

Figure 5F

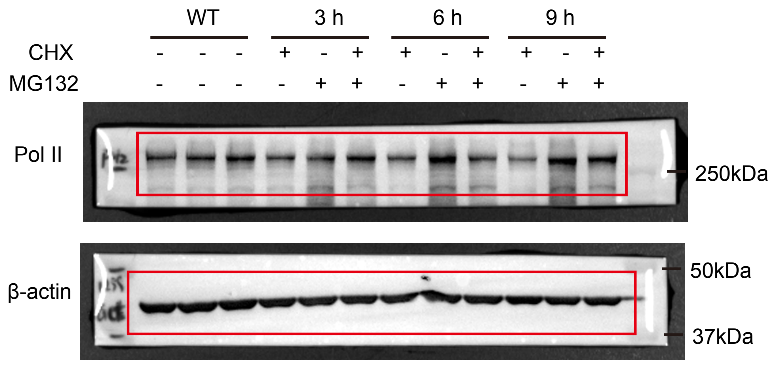

Figure S2 F

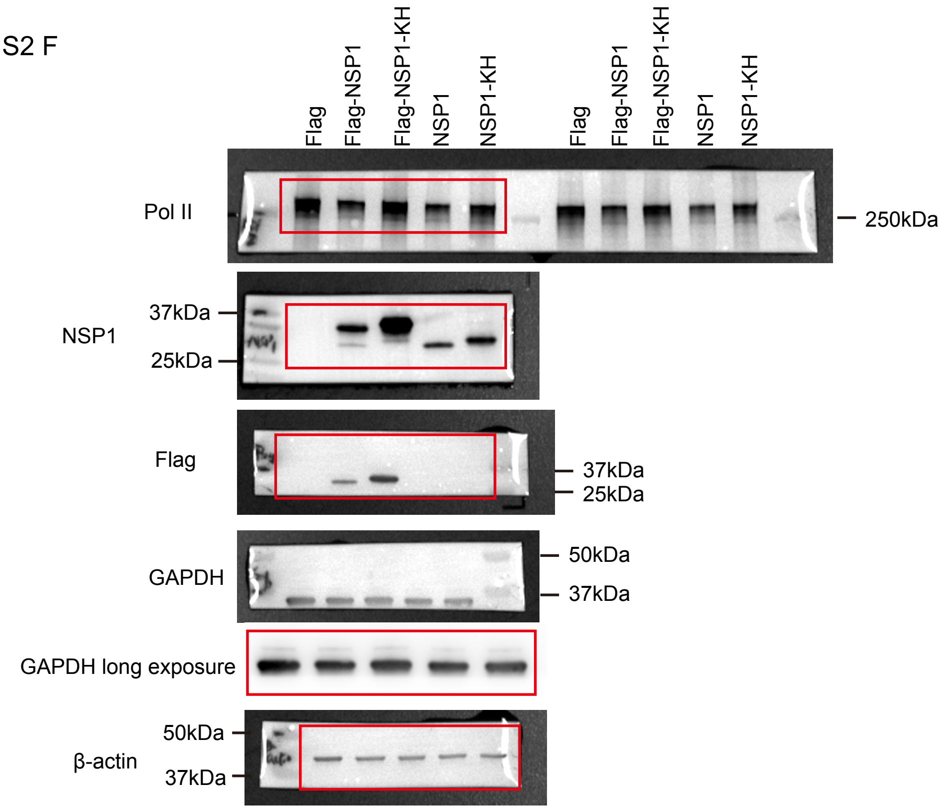

Figure S2 G

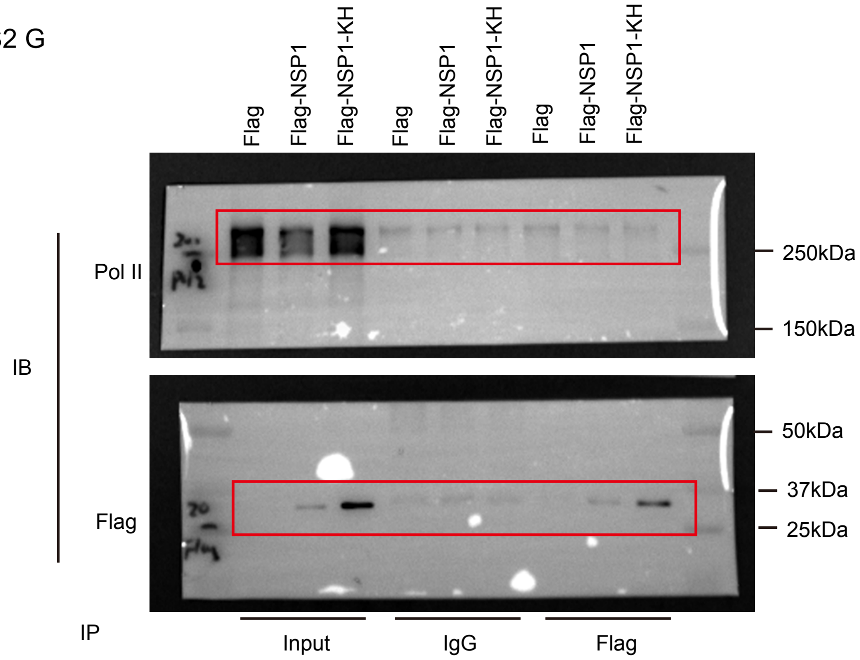

Figure S2 H

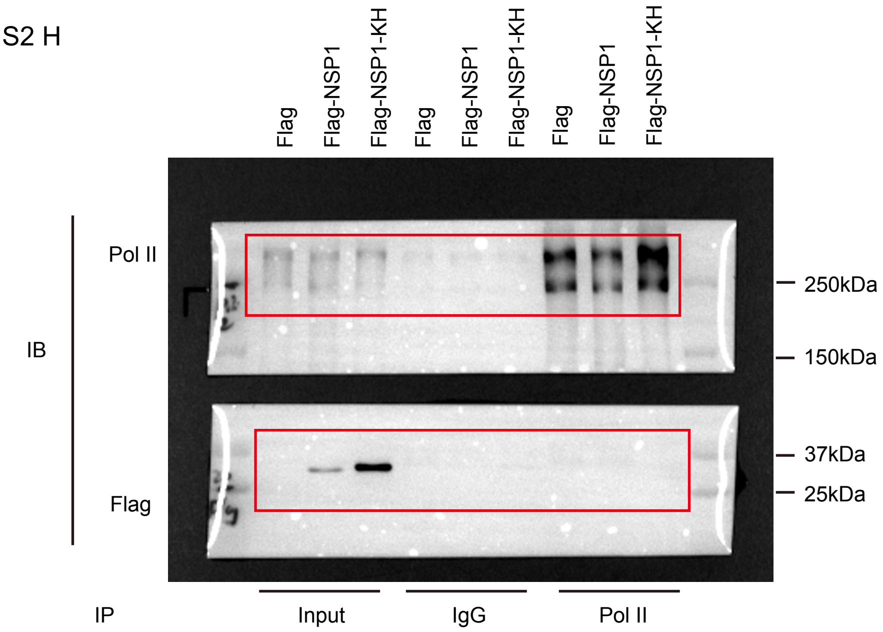

Supplement: Data S2. The raw data of western blots [file mmc3.pdf]
